# Bis(oxalato)borate Salts as Safe and Fluorine‐Free Alternatives to Conventional Supporting Electrolytes for Organic Electrosynthesis

**DOI:** 10.1002/cssc.202502655

**Published:** 2026-04-05

**Authors:** Anton Scherkus, Zeng He, Robert Francke

**Affiliations:** ^1^ Leibniz Institute for Catalysis Rostock Germany

**Keywords:** bis(oxalato)borate, electrosynthesis, organic electrochemistry, supporting electrolyte, sustainability

## Abstract

Supporting electrolytes are indispensable in organic electrosynthesis, since they provide the ionic conductivity required for closing the electrical circuit. In combination with organic solvents, lithium or tetraalkylammonium salts of perchlorate, tetrafluoroborate, and hexafluorophosphate are typically employed. Although these salts exhibit good solubility and high electrochemical stability, there are known issues with chemical stability, safety, and sustainability. Therefore, problems are to be expected when transferring lab‐scale innovations to industrial application, which is why sustainable and affordable alternatives with comparative performance are needed. In this context, we explored the use of commercially available lithium bis(oxalato)borate as potential candidate. In the present work, we compare the (electro)chemical properties to the standard salts in typical reaction media and characterize the performance in electrosynthesis using three different test cases. It was found that LiBOB can compete with LiClO_4_, LiPF_6_, and LiBF_4_ in terms of electrochemical stability and ionic conductivity. Anodic synthesis of diaryliodonium compounds, cathodic ketone reduction, and TEMPO‐mediated oxidation of alcohols were selected as test reactions, showing that product yields are comparable to the ones obtained with conventional salts. Analysis of the post‐electrolysis reaction mixtures showed that the BOB anion remains intact throughout electrolysis when aprotic electrolytes are used.

## Graphical abstract

Lithium bis(oxalato)borate (LiBOB) was established as a new tool in the repertoire of the synthetic organic electrochemist. As a sustainable alternative to conventional supporting electrolytes, LiBOB shows high stability and ionic conductivity while in test reactions, product yields are comparable to standard electrolyte systems.

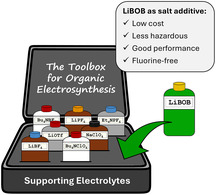

## 1 Introduction

Organic electrosynthesis is generally considered as an inherently sustainable method [1, 2, 3], which aligns well with the 12 principles of green chemistry [4]. Indeed, the methodology offers the chance to reduce energy consumption, waste production, and hazards in comparison with conventional organic syntheses by replacing reagents with electricity [5]. However, aside from these opportunities, there are also a number of challenges along the path to truly effective and sustainable electrosyntheses [6, 7, 8]. One of these challenges is the discharge reaction at the counter electrode, which can lead to the formation of stoichiometric amounts of waste. Another problem is the frequent use of solvents such as fluorinated alcohols, DMF, CH_2_Cl_2_, and CH_3_CN [9], which have excellent electrochemical properties but undermine the promise of sustainable electrosynthesis [10]. The solvent problem has already been recognized and tackled in first systematic studies on alternative solvents [11, 12, 13]. The need to use a supporting electrolyte poses a further challenge, as the salt additive may complicate the separation of the product mixture and constitutes a source of waste. In various studies, the supporting electrolyte issue has been addressed by developing innovative concepts for simplifying separation and recycling, including particle‐immobilized ionic species [14, 15], polyelectrolytes [16], and multi‐functional salts [17, 18, 19, 20, 21]. In most synthetic scenarios, however, conventional salts will likely remain the supporting electrolytes of choice.

Due to their good solubility in organic solvents and favorable electrochemical properties, lithium or tetraalkylammonium salts of weakly coordinating anions such as perchlorate, hexafluorophosphate, and tetrafluoroborate are typically used as supporting electrolytes [9]. Among the mentioned species, particularly the anions exhibit issues regarding safety and sustainability. For example, hexafluorophosphate has significant shortcomings in terms of thermal and chemical stability. LiPF_6_ decomposes above 70°C when dissolved in polar aprotic solvents [22], above 134°C in dry anhydrous state [23], as well as in the presence of water, whereby hydrolysis is accompanied by the release of HF. The lack of stability is reflected by a recent study on the anodic synthesis of diaryliodonium compounds using LiPF_6_ as salt additive, wherein decomposition of PF_6_^–^ into mixed phosphates (PO_3_F^2−^ and PO_2_F_2_^−^) was demonstrated [24]. Perchlorate is an equally problematic candidate since, although it is electrochemically stable, it decomposes readily in the presence of organic compounds when heated, which may even result in explosion [25]. The tetrafluoroborate ion, on the other hand, shows higher chemical stability but dissociates only poorly in combination with alkali metal cations in organic media [22]. From this perspective, the search for and testing of an easily accessible, stable, and safe substitute is essential for the development of sustainable electrochemical syntheses.

When searching for a possible replacement for the problematic weakly coordinating anions, it is worth considering electrochemical energy storage systems, in which the electrolyte is composed of an organic solvent and electrochemically stable supporting electrolytes. In this context, lithium bis(oxalato)borate (LiBOB) is a particularly promising candidate, which has been extensively studied with respect to the use in lithium‐ion batteries (LIBs) [22, 26]. Compared to the standard salt LiPF_6_, LiBOB exhibits promising features such as higher thermal stability (thermal decomposition above 293°C in the dry anhydrous state) [27], higher overall safety, a comparable electrochemical window, and a stabilizing effect on the solid electrolyte interface (SEI) between graphite electrodes and the electrolyte [28, 29, 30]. Moreover, the salt has been commercialized and is therefore easily accessible and affordable. All these promising characteristics have prompted us to conduct a systematic study on the use of LiBOB in synthetic organic electrochemistry. The present work focuses on analyzing the key‐physicochemical properties (conductivity and electrochemical stability) in solvents commonly used in electrosynthesis and evaluating the performance during electrolysis. For the latter, three representative test reactions were selected, i.e., a direct anodic oxidation (synthesis of diaryliodonium compounds), a mediated process (TEMPO‐catalyzed oxidation of alcohols), and a direct cathodic reduction (conversion of benzophenone to diphenylmethanol).

## 2 Results and Discussion

The primary role of the supporting electrolyte during electrolysis is to close the electrical circuit, which is why high solubility and good specific conductivity are desirable properties. In some cases, the supporting electrolyte plays additional roles, such as adjusting a specific pH value, acting as a sacrificial agent for discharge at the counter electrode (depolarizer), reacting with electrochemically generated intermediates (reactant), or catalyzing reactions (mediator) [17]. In these cases, the supporting electrolyte must meet additional specific requirements tailored to the reaction. In most cases, however, a rather passive role in relation to the reaction is desired, demanding for chemical and electrochemical inertness. Section 2.1 focuses on chemical stability, electrochemical window, and ionic conductivity as key properties, while Section 2.2 describes the performance in specific synthetic applications.

### 2.1 Stability and Conductivity

**FIGURE 1:**
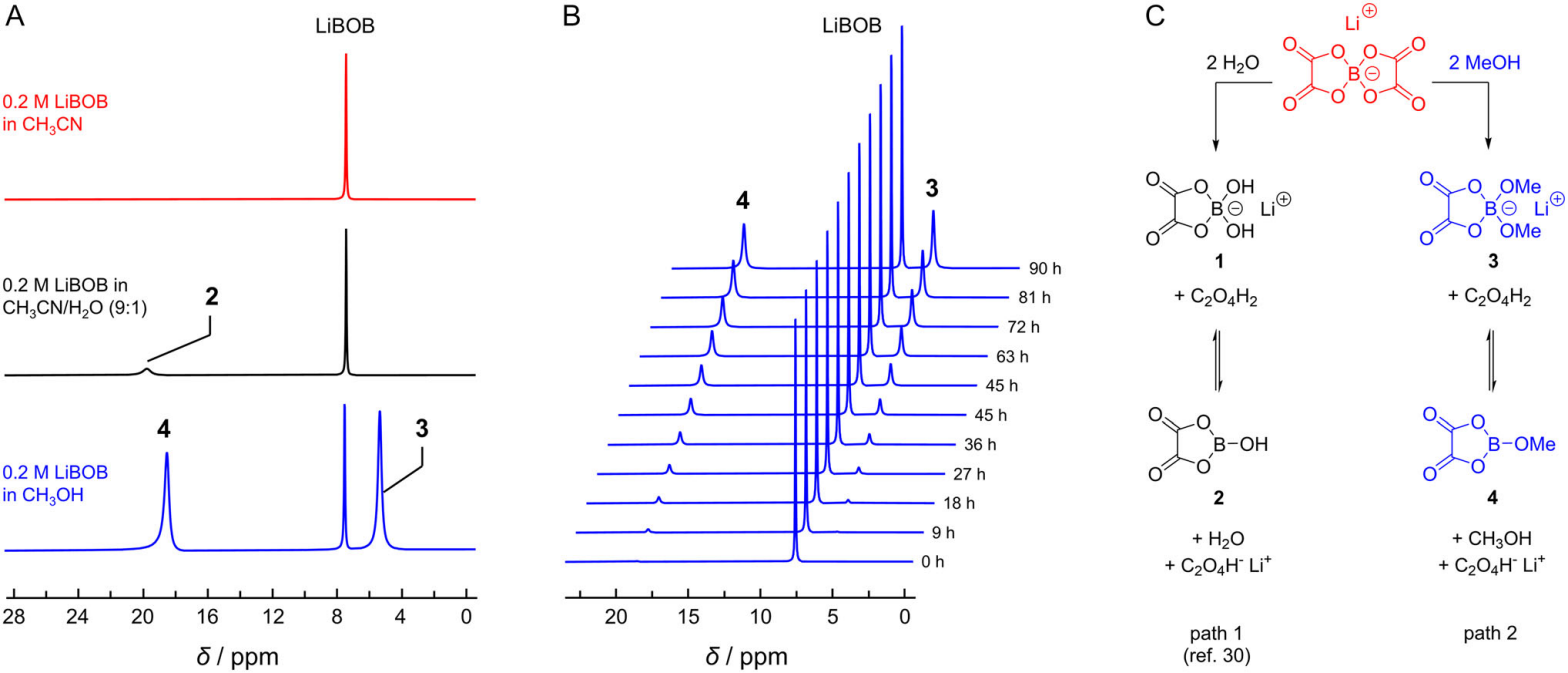
(A) ^11^B NMR spectra of 0.2 M LiBOB solutions in CH_3_CN, CH_3_CN/H_2_O 9:1 (*v*/*v*), and CH_3_OH recorded after aging for 96 h at room temperature. (B) Time‐resolved ^11^B NMR analysis of the degradation of 0.2 M LiBOB in MeOH. (C) Degradation mechanism in MeOH (path 2) proposed in analogy to the hydrolysis of LiBOB in H_2_O (path 1) described in Ref. [30].

To investigate the chemical stability of LiBOB in media relevant for electrosynthesis, 0.2 M solutions in CH_3_CN, CH_3_CN/H_2_O 9:1 (*v*/*v*), and CH_3_OH were stored for 96 h at room temperature, followed by ^11^B NMR analysis (Figure 1A). When handling LiBOB, it should be noted that the solid material exhibits a certain degree of hygroscopicity. For example, it was reported that under atmospheric conditions at room temperature (relative humidity: 70%), anhydrous LiBOB completely converts into LiBOB·H_2_O in approximately 30 min [31]. Consequently, handling in the air should proceed rapidly to minimize exposure to moisture. In our experience, however, this hygroscopic behavior did not pose a particular problem in contrast to other salts such as LiClO_4_ or LiOTf, which absorb moisture much faster and can even form hydrogels when exposed to air.

In pure CH_3_CN, aside from the peak at 7 ppm associated with the BOB anion, no traces of decomposition were observed. In CH_3_CN/H_2_O 9:1 (*v*/*v*), however, a minor amount of a decomposition product was detected based on a small peak at 19.8 ppm. An even more pronounced change was observed in MeOH. Thus, two broad signals emerged at 18.5 and 5.4 ppm, suggesting decomposition (methanolysis) of the bis(oxalato)borate anion. A time‐resolved ^11^B NMR study showed that the signals are already detectable after 9 h and continuously increase in intensity (Figure 1B).

The hydrolysis of bis(oxalato)borate has been previously studied in dialkyl carbonate/water mixtures as well as in pure water [30]. In water, the formation of two peaks at 20 and 4.5 ppm was observed, similar to the results in Figure 1A. These signals were assigned to boronic acid derivative **1** (20 ppm) and borate **2** (4.5 ppm), as indicated Figure 1C (path 1). Temperature‐dependent ^11^B NMR measurements showed that the newly formed species are in continuous exchange. In analogy to BOB hydrolysis, we propose the formation of **3** and **4** according to path 2 (Figure 1C) for the decomposition of BOB in methanol. This methanolysis pathway is confirmed both by the similar chemical shifts of the two newly appearing ^11^B NMR signals and by a signal at *m*/*z* = 161 obtained by ESI(‐) MS analysis of the solution, which can be assigned to species **3**.

To put the observed behavior of the BOB anion into perspective, it should be noted that other seemingly inert anions can undergo solvolysis as well. For example, it is known that the hexafluorophosphate anion decomposes in presence of water or alcohols to give mixed phosphate species [32, 33, 34]. For comparison, LiBF_4_ and LiPF_6_ were investigated in the same manner. While no signs of decomposition were observed in CH_3_CN after 90 h, signals associated with solvolysis products of BF_4_^–^ and PF_6_^–^ appeared in CH_3_CN/H_2_O (9:1) and CH_3_OH (see the SI). However, in contrast to LiBOB, degradation occurred to a much smaller extent. This finding highlights that the BOB anion is more prone to hydrolysis and methanolysis compared to BF_4_^–^ and PF_6_^–^, aligning with an earlier kinetic study of the hydrolysis of LiBF_4_ and LiBOB [35].

**FIGURE 2:**
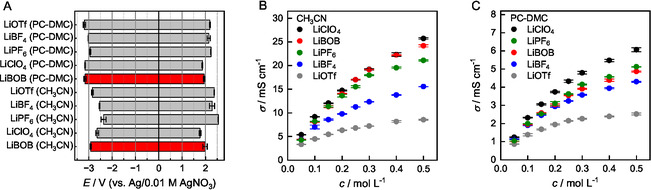
Comparison between the electrochemical properties of LiBOB, LiClO_4_, LiPF_6_, LiBF_4_, and LiOTf. (A) Electrochemical windows of 0.1 M solutions in PC‐DMC (4:1, *m*/*m*) and CH_3_CN at room temperature. WE = glassy carbon, *v* = 10  mV s^–1^. Definition of the stability limit using threshold current density of 0.1 mA  cm^−2^. (B) Ionic conductivity in CH_3_CN at 25°C. (C) Ionic conductivity in PC‐DMC (4:1, *m*/*m*) at 25°C.

The electrochemical windows of 0.1 M LiBOB solutions in PC–DMC (4:1) and CH_3_CN were determined by cyclic voltammetry (CV) at 10 mV  s^−1^, whereby a current density of │0.1 mA cm^–2^│ was defined as the stability limit. The results are summarized in the bar diagram in Figure 2A (red bars) and compared to the electrochemical windows of LiClO_4_, LiPF_6_, LiBF_4_, and LiOTf (grey bars). The potentials read out at the threshold current density form the outer edges of the bars. Both in PC–DMC (4:1) and CH_3_CN, a stability range of approximately 5 V is available, allowing for anodic and cathodic transformations even at elevated potentials. While in PC–DMC, both the position and the width of the window is comparable to the ones of the reference salts, more pronounced differences can be observed in CH_3_CN. In the latter solvent, LiBOB exhibits a stability window that is more than 0.5 V wider than that of LiClO_4_. The stability range in CH_3_CN is roughly comparable to LiBF_4_, LiPF_6_, and LiOTf, but shifted slightly in the cathodic direction.

In CH_3_CN, the ionic conductivities (*σ*) measured at 25°C follow the trend LiClO_4 _ ≈  LiBOB  >  LiPF_6 _ >  LiBF_4 _ >  LiOTf. For example, a 0.5 M LiBOB solution features a conductivity of nearly 25 mS cm^–1^, an excellent value that offers favorable conditions for electrosynthetic applications. A similar trend with respect to the five salts can also be observed in PC‐DMC (4:1), albeit with *σ* values that are approx. five times lower due to the increased viscosity of the medium. Taken together, LiBOB can compete with the three reference salts in terms of electrochemical stability and clearly outperforms both LiBF_4_ and LiOTf with respect to ionic conductivity.

The conversion of bis(oxalato)borate in methanol highlighted in Figure 1 was investigated towards an alteration of both the electrochemical window and conductivity. With a window of 2.7 V, the electrochemical stability of a freshly prepared 0.1 M solution of LiBOB in CH_3_OH is already significantly smaller (see the SI). Interestingly, the conversion of the BOB anion to methanolysates **3** and **4** has a noticeable effect, as the window is further diminished to 2.2 V after 96 h of aging. Methanolysis of BOB also influences the ionic conductivity, which decreases from initially 10.0 to 6.3 mS  cm^−1^ after 96 h. Based on these findings, the use of BOB salts in the presence of proton donors appears less promising. However, successful use in protic media cannot be ruled out at this point.

### 2.2 Synthetic Studies

**FIGURE 3:**
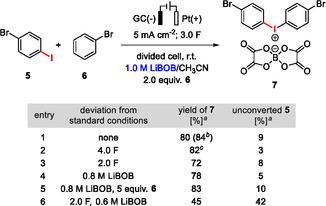
Optimization of the electrosynthesis of diaryliodonium salt **7** using LiBOB as supporting electrolyte and anion source. Batch size: 1.0 mmol. ^a^Yields determined by ^1^H NMR spectroscopy using an internal standard. ^b^Isolated yield. ^c^Dark coloration of the solution and formation of side products observed.

With promising electrochemical properties at hand, the behavior of LiBOB was investigated using the synthesis of diaryliodonium salts in acetonitrile as a representative case of an anodic oxidation. Diaryliodonium compounds have received growing interest over the last decades as metal‐free, easy‐to‐handle, and efficient arylation reagents [36, 37, 38]. A variety of conventional methods are available for synthesizing these compounds, many of them involving the use of problematic reagents [39, 40]. Electrochemical methods have been developed as sustainable alternatives, which, however, only allow a limited selection of counterions in the product [41, 42, 43, 44]. Since the counterion plays a crucial role in terms of the reactivity of the iodonium species and the yield of the arylation reaction [45], we recently developed an ‘anion flexible’ electrochemical approach [24] which served as the starting point for this study. The method involves the use of a Li^+^ salt of the anion of choice as supporting electrolyte in acetonitrile, in combination with a Pt anode and a glassy carbon cathode. A divided cell is necessary to prevent the reduction of the diaryliodonium compound at the cathode. The coupling between *p*‐bromoiodobenzene (**5**) and bromobenzene (**6**) to furnish diaryliodonium bis(oxalato)borate salt **7** was selected as an example. The results are summarized in Figure 3.

In our recent study, ClO_4_^–^, BF_4_^–^, OTf^–^, NTf_2_^–^ and PF_6_^–^ were introduced as counterions in analogous conversions, whereby the optimized yields of the corresponding diaryliodonium salts ranged from 78% to 99% [24]. Based on these results, the electrosynthesis was optimized for the use of LiBOB as a supporting electrolyte and anion source. The optimal conditions include 2.0  equiv. **6**, a current density of 5.0  mA  cm^–2^, 3.0 transferred charge equivalents per mole of **5**, and a LiBOB concentration of 1.0 M. Under these conditions, **7** could be isolated in a 84% yield (80% ^1^H NMR yield, table in Figure 3, entry 1). Although an attempt with a higher amount of charge slightly increased the NMR yield, dark coloration of the solution and formation of by‐products was observed (entry 2). Further attempts to reduce the amount of charge and the LiBOB loading resulted in inferior yields (entries 3–6). Noteworthy, the ^11^B NMR spectrum of the post‐electrolysis electrolyte solution shows only the BOB signal at 7.5 ppm (see the SI), highlighting the anion stability under the reaction conditions.

**FIGURE 4:**
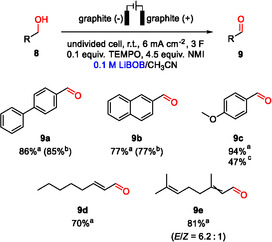
TEMPO‐mediated oxidation of alcohols using LiBOB as supporting electrolyte. Batch size: 1.0 mmol. ^a^Yields determined with ^1^H NMR spectroscopy using an internal standard. ^b^Isolated yields. ^c^Yield obtained when using 0.04 M NaClO_4_ under otherwise identical conditions [11].

In the next step, the use of LiBOB was tested in the anodic TEMPO‐catalyzed alcohol oxidation (Figure 4) based on a protocol recently developed by us [11]. The reported approach allows for electrolysis in an undivided cell under galvanostatic conditions with moderate catalyst loading, a significant progress with respect to sustainability and effectiveness compared to the standard protocol [46]. A remaining issue with our method was the use of NaClO_4_ as salt additive with the associated risks discussed in the introduction. The results shown in Figure 4 illustrate that frequently used perchlorate salts can be replaced by LiBOB and that useful results can be achieved this way.

All TEMPO‐mediated reactions within this study were carried out in CH_3_CN. The optimized conditions reported in Ref. [11], namely a current density of 6  mA  cm^–2^, 4.5  equiv. *N*‐methylimidazole (NMI, used as proton scavenger), and 0.1  equiv. TEMPO, served as an entry point, from which a screening of the supporting electrolyte loading was carried out. For this purpose, the synthesis of **9a** from the corresponding alcohol was used as a test case (see Table S4). The results show a continuous increase in yield with salt concentration, with a good balance between material input and yield found at 0.1 M LiBOB. Under these conditions, **9a** was obtained in an 85% isolated yield (86% ^1^H NMR yield). A brief study on the substrate scope shows that the method can be applied to other benzylic (**9b** and **9c**) and allylic alcohols (**9d** and **9e**), whereby good to excellent ^1^H NMR yields were achieved (70%–94%). Due to the volatility of compounds **9c**–**e**, product isolation was not pursued in these cases. Interestingly, the ^1^H NMR yield obtained for **9c** is significantly lower when 0.04 M NaClO_4_ is used instead of LiBOB under otherwise identical conditions (47% instead of 94%). An unexpected *E*/*Z* isomerization occurred upon conversion of isomerically pure geraniol, leading to an *E*/*Z* ratio of ∼6:1 for product **9e** (for details, see the SI). With respect to anion stability under electrolysis conditions, no signs of degradation were observed when subjecting the post‐electrolysis reaction mixture to ^11^B NMR spectroscopy (see the SI).

**FIGURE 5:**
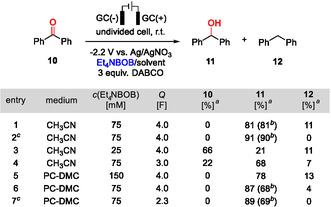
Direct cathodic reduction of benzophenone under potentiostatic conditions using Et_4_NBOB as supporting electrolyte. Standard batch size: 1 mmol. 100 µL CH_3_OH added per mmol of **10**. ^a^Yields determined by ^1^H NMR spectroscopy using 1,3,5‐trimethoxybenzene as an internal standard. ^b^Isolated yield. ^c^Batch size: 5 mmol.

The conversion of benzophenone (**10**) to diphenyl methanol (**11**) served as a test case for a direct (uncatalyzed) cathodic reduction (Figure 5). Several electrochemical procedures have been reported over the last years [11, 13, 47], and a comparison of the results with the present approach is provided in Table 1. The reported methods differ mainly in the choice of solvent, with DMF [47], Cyrene [13], and PC–DMC (1:4) [11] being used. On the other hand, all previous procedures have in common that glassy carbon electrodes, Bu_4_NBF_4_ supporting electrolyte, and an undivided cell were used in combination with 1,4‐diazabicyclo [2.2.2]octane (DABCO) as anodic depolarizer. Since Li^+^ salts have been reported to promote pinacol coupling of **10** [13], we opted for using Et_4_NBOB instead of LiBOB. The studies were carried out in CH_3_CN (table in Figure 5, entries 1–4) and in PC–DMC (4:1, entries 5–7), respectively. In each case, a small amount of proton donor was added (100 µL CH_3_OH per mmol **10**). Compared to Ref. [11], a higher proportion of PC was used, since Et_4_NBOB is not sufficiently soluble in PC–DMC (1:4). Since Et_4_NBOB is not commercially available, it was prepared from LiBOB *via* cation exchange (see the SI). The complete results of the reaction optimization are shown in the SI, while only the key results are discussed here.

**TABLE 1:** Comparison between selected conversions of 10 to 11 with respect to reaction media, scale, and green metrics (details for calculations are provided in the SI).

	This work (cathodic reduction)	This work (cathodic reduction)	Ref. [47] (cathodic reduction)	Ref. [11] (cathodic reduction)	Ref. [48] (reduction with NaBH_4_)
Supporting electrolyte	75 mM Et_4_NBOB	75 mM Et_4_NBOB	50 mM Bu_4_NBF_4_	120 mM Bu_4_BF_4_	—
Solvent	CH_3_CN	PC‐DMC (4:1)	DMF	PC‐DMC (1:4)	EtOH
Batch size (mmol)	5.0	5.0	0.3	5.0	5.5
Depolarizer/reductant (equiv.)	3.0	3.0	3.0	2.2	3.6
*c*(substrate) (M)	0.5	0.5	0.06	0.5	0.11
Isolated yield (%)	90	69	79	82	>99
PMI	17.7	33.0	114.2	17.9	40.2
PMI_RRC_	3.6	4.6	5.4	3.4	1.1
PMI_solv_	14.1	28.4	108.8	14.5	39.1

In acetonitrile, optimal results were achieved after passing 4.0 F and at a Et_4_NBOB concentration of 75 mM (table in Figure 4, entry 1). Scaling up the batch size from 1 to 5 mmol even led to an increase in the isolated yield from 81% to 90% (entry 2). Attempts to reduce the supporting electrolyte concentration (entry 3) and the number of charge equivalents (entry 4) resulted in lower yields. In PC–DMC (4:1), the best results were also achieved at *c*(Et_4_NBOB) = 75 mM (entries 5 and 6). While the ^1^H NMR yields are comparable to those achieved in CH_3_CN, the isolation of **11** resulted in minor material losses due to the high boiling point of PC (68% yield, entry 6). The separation procedure involved bulb‐to‐bulb distillation at high vacuum (for details, see the SI). Scaling up the batch from 1 mmol to 5 mmol resulted in an almost identical yield (69%, entry 7). Interestingly, small amounts of deoxygenation product **12** are formed as by‐products in several cases. The comparison in Figure 5 shows that Et_4_NBOB is compatible with both reaction media and produces similar results. While PC and DMC feature superior solvent sustainability ratings [10], separating the product mixture on a laboratory scale is significantly easier with CH_3_CN. Noteworthy, the BOB anion was completely converted to **3** and **4** by reaction with the CH_3_OH proton donor after passing two F per mole starting material, as evidenced by ^11^B NMR spectroscopy of the reaction mixture (see the SI). Compared to the methanolysis profile presented in Figure 1B, passing electric current seems to promote BOB degradation, possibly *via* electrochemically induced pH gradients.

Figure 5 shows that the use of Et_4_NBOB renders useful results both in CH_3_CN and in PC–DMC. To make further assertions on efficiency and sustainability of the reaction, the isolated yields and process mass intensities (PMI) are compared to literature examples (Table 1; for details, see the SI). The PMI value describes the ratio between the mass of all components employed (reactants, reagents, catalysts, and solvent) and the mass of the isolated product. Further metrics summarized in Table 1 are PMI_solv_ (mass of solvent vs. mass of isolated product) and PMI_RRC_ (total mass of reactants, reagents and catalysts vs. mass of product). In comparison to the previously reported cathodic reduction of **10** in DMF [47], the PMI values obtained in this study are considerably better, which can be ascribed both to an improved yield and to a higher concentration of the starting material.

Compared with the method recently reported in Ref. [11], a slightly better PMI was achieved in CH_3_CN, which results from a reduced supporting electrolyte loading and an improved product yield. With respect to a reported chemical reduction using NaBH_4_ [48], our PMI values are significantly lower, which can be ascribed mostly to a higher concentration of starting material. However, our PMI_RRC_ value is considerably worse, which results from the use of supporting electrolyte and depolarizer (DABCO), the latter having a significantly higher molar mass compared with the chemical reductant NaBH_4_. The results in Table 1 challenge the often postulated ‘inherent greenness’ of electrosynthesis and clearly show how much sustainability and effectiveness depend on the way the method is used. The results highlight that, in addition to employing sustainable reaction components, further goals of reaction optimization should be to achieve highest possible substrate concentrations, to reduce the supporting electrolyte loading, and to realize resource‐saving discharge reactions at the counter electrode.

## 3 Conclusion

In summary, our study reveals promising features of BOB salts, which qualify them as alternatives to conventional supporting electrolytes in organic electrochemistry. The electrochemical window in polar aprotic reaction media can be rated as excellent, providing sufficient stability to enable both challenging anodic oxidations and cathodic reductions. With respect to ionic conductivity, commercially available LiBOB provides competitive values and even outperforms LiBF_4_ and LiOTf, thereby enabling electrolysis at moderate cell voltage.

Unlike the frequently used perchlorates, there are no particular hazards associated with handling of LiBOB. Although the tendency toward solvolysis is more pronounced compared to hexafluorophosphate and tetrafluoroborate salts, it does not lead to the release of toxic hydrogen fluoride. During the TEMPO‐catalyzed electrochemical oxidation of alcohols and the anodic synthesis of diaryliodonium compounds, the BOB anion remains stable even upon passing over‐stoichiometric amounts of charge, while the yields are comparable to those obtained using conventional supporting electrolytes.

Potential issues may occur in protic media due to solvolysis of the BOB anion. Thus, during cathodic ketone reduction, methanolysis was observed when using CH_3_OH as a proton donor. Even though there was no adverse effect on yield in this case, decomposition can in principle lead to problems when recycling the supporting electrolyte.

In view of the promising results described herein, we encourage readers with a focus on organic electrochemistry to include LiBOB in supporting electrolyte screenings for future reaction developments. Further investigations into the respective tetraalkylammonium salts are needed, from which we expect an expansion of the scope of application for BOB‐based supporting electrolytes.

## 4 Supporting Information

Additional supporting information can be found online in the Supporting Information section. **Supporting Fig. S1**: Time‐dependent ^11^B NMR analysis of a 0.2 M solution of LiBOB in CH_3_CN. Top: Full spectra. Bottom: Enlarged sections illustrating the stability of BOB. **Supporting Fig. S2**: Time‐dependent ^11^B NMR analysis of a 0.2 M solution of LiBOB in CH_3_CN/H_2_O (9:1). Top: Full spectra. Bottom: Enlarged sections illustrating BOB degradation. **Supporting Fig. S3**: Time‐dependent ^11^B NMR analysis of a 0.2 M solution of LiBOB in CH_3_OH. Top: Full spectra. Bottom: Enlarged sections illustrating BOB degradation. **Supporting Fig. S4**: Time‐dependent ^11^B NMR analysis of a 0.2 M solution of LiBF_4_ in CH_3_CN. Top: Full spectra. Bottom: Enlarged sections illustrating the stability of the BF_4_^−^ species. **Supporting Fig. S5**: Time‐dependent ^11^B NMR analysis of a 0.2 M solution of LiBF_4_ in CH_3_CN/H_2_O (9:1). Top: Full spectra. Bottom: Enlarged sections illustrating formation of a degradation product. **Supporting Fig. S6**: Time‐dependent ^11^B NMR analysis of a 0.2 M solution of LiBF_4_ in CH_3_OH. Top: Full spectra. Bottom: Enlarged sections illustrating formation of a degradation product. **Supporting Fig. S7**: Time‐dependent ^31^P NMR analysis of a 0.2 M solution of LiPF_6_ in CH_3_CN. Top: Full spectra. Bottom: Enlarged sections illustrating the presence of a degradation product. **Supporting Fig. S8**: Time‐dependent ^31^P NMR analysis of a 0.2 M solution of LiPF_6_ in CH_3_CN/H_2_O (9:1). Top: Full spectra. Bottom: Enlarged sections illustrating formation of degradation products. **Supporting Fig. S9**: Time‐dependent ^31^P NMR analysis of a 0.2 M solution of LiPF_6_ in CH_3_OH. Top: Full spectra. Bottom: Enlarged sections illustrating the presence of degradation products. **Supporting Fig. S10**: Voltammetric analysis of 0.1 M supporting electrolyte solutions in acetonitrile. A) Linear sweeps in the reductive regime. B) Linear sweeps in the oxidative regime. The shown LSVs are the forward scans of the third cycles taken from CV measurements recorded under the conditions described above. **Supporting Fig. S11**: Voltammetric analysis of 0.1 M supporting electrolytes in PC/DMC (4:1 *w*/*w*). A) Linear sweeps in the reductive regime. B) Linear sweeps in the oxidative regime. The shown LSVs are the forward scans of the third cycles taken from CV measurements recorded under the conditions described above. **Supporting Fig. S12**: Voltammetric analysis of a fresh (blue line) and aged (black line, 90 h at room temperature) 0.1 M solution of LiBOB in MeOH. A) Linear sweeps in the reductive regime. B) Linear sweeps in the oxidative regime. The shown LSVs are the forward scans of the third cycles taken from CV measurements recorded under the conditions described above. **Supporting Fig. S13**: Monitoring the stability of the BOB anion during electrochemical synthesis of diaryl iodonium salt **7**. 11B NMR spectra of a freshly prepared reaction mixture (left) and after passing 4 F per mole **5** (right, for electrolysis conditions, see general procedure). Electrolyte aliquots were diluted with CD_3_CN for NMR analysis. **Supporting Fig. S14**: Monitoring the stability of the BOB anion during TEMPO‐catalyzed alcohol oxidation. Exemplary 11B NMR spectra of a freshly prepared reaction mixture (left) and after passing 3 F per mole alcohol (right, for electrolysis conditions, see general procedure). Aliquots of the electrolyte solution were diluted with CD_3_CN for NMR analysis. **Supporting Fig. S15**: Monitoring the conversion of BOB during cathodic reduction of benzophenone using MeOH as a proton donor. Exemplary 11B NMR spectra of a freshly prepared reaction mixture (left) and after passing 2 F per mole **10** (right, for electrolysis conditions, see general procedure). Electrolyte aliquots were diluted with CD_3_CN for NMR analysis. **Supporting Fig. S16**: 1H NMR spectrum of crude electrolysis mixture from TEMPO‐mediated oxidation of *p*‐methoxybenzyl alcohol (inset: characteristic region of aldehyde proton signals). **Supporting Fig. S17**: 1H NMR spectrum of crude electrolysis mixture from TEMPO‐mediated oxidation of (*E*)‐2‐octen‐1‐ol (inset: characteristic region of aldehyde proton signals). **Supporting Fig. S18**: 1H NMR spectrum of crude electrolysis mixture from TEMPO‐mediated oxidation of geraniol (inset: characteristic region of aldehyde proton signals). **Supporting Fig. S19**: Gas chromatogram (top) and corresponding mass spectra (bottom) obtained from analysis of the reaction mixture after completed TEMPO‐catalyzed electro‐conversion of geraniol. **Supporting Table S1**: Anodic and cathodic stability limits (*E*_red_ and *E*_ox_) determined at 10 mV  s^−1^ using 0.1 M solutions of the respective salt (see Figure S10 and Figure S11). **Supporting Table S2**: Conductivity values determined for LiBOB and reference salts at 25°C in acetonitrile for various concentrations. **Supporting Table S3**: Conductivity values determined for LiBOB and reference salts at 25°C in PC/DMC (4:1, *w*/*w*) for various concentrations. **Supporting Table S4**: Optimization of the LiBOB concentration under the conditions described in the general procedure (yields determined by 1H NMR spectroscopy using 1,3,5‐trimethoxybenzene as internal standard). **Supporting Table S5**: Optimization of the cathodic reduction of benzophenone (**10**) to diphenylmethanol (**11**) in an undivided cell at −2.2 V vs. Ag/AgNO_3_. Diphenylmethane (**12**) occurs as a side product. Isolated yields are added in parentheses (entries 1, 2, 11, and 12). **Supporting Table S6**: Summary of the parameters used for calculation of PMI, PMI_RRC_, and PMI_solv_ values for the conversion of **10** to **11** on a 5.0 mmol scale in acetonitrile (see Table 1 in manuscript).^10^ **Supporting Table S7**: Summary of the parameters used for calculation of PMI, PMI_RRC_, and PMI_solv_ values for the conversion of **10** to **11** on a 5.0 mmol scale in PC/DMC (4:1) (see Table 1 in manuscript).^10^ **Supporting Table S8**: Summary of the parameters used for calculation of PMI, PMI_RRC_, and PMI_solv_ values for electrochemical conversion of **10** to **11** in DMF.^11^ **Supporting Table S9**: Summary of the parameters used for calculation of PMI, PMI_RRC_, and PMIsolv values for non‐electrochemical ketone reduction in ethanol using NaBH_4_ as reducing reagent.^12^

## Funding

This work was supported by the Deutsche Forschungsgemeinschaft (460129901 and 441548530) and the Chinese Scholarship Council (doctoral stipend).

## Conflicts of Interest

The authors declare no conflicts of interest.

## Supporting information

Supplementary Material

## Data Availability

The data that support the findings of this study are available in the supplementary material of this article.
