# Supplementary material for: Bis(oxalato)borate Salts as Safe and Fluorine‐Free Alternatives to Conventional Supporting Electrolytes for Organic Electrosynthesis
Source: ChemSusChem. 2026 Apr 5;19(7):e202502655. doi: 10.1002/cssc.202502655 (PMC13050548; doi:10.1002/cssc.202502655)
Supplement: Supplementary file 1 — Supplementary Material [file CSSC-19-e202502655-s001.pdf]

Supporting Information

Bis(oxalato)borate Salts as Safe and Fluorine-free Alternatives to Conventional Supporting Electrolytes for Organic Electrosynthesis

Anton Scherkus, Zeng He, Robert Francke*

Leibniz Institute for Catalysis, Albert-Einstein-Str. 29a, 18059 Rostock, Germany

*Corresponding author. E-mail: robert.francke@catalysis.de

Content

1. General remarks S2
2. (Electro)chemical properties of LiBOB and reference salts S3
 2.1 Chemical stability S3
 2.2 Electrochemical stability S12
 2.3 Ionic conductivity S15
3. Preparative-scale electrolysis S18
 3.1 Electrosynthesis of diaryliodonium compounds S18
 3.2 TEMPO-mediated oxidation of alcohols S19
 3.3 Cathodic reduction of benzophenone S21
4. NMR spectra S23
 4.1 Analysis of isolated compounds S23
 4.2 Quantification of product yields by ^{1}H NMR spectroscopy S30
5. Green metrics estimated for the synthesis of **11** S33

1. General remarks

^{1}H and ^{13}C NMR spectra were recorded using an AVANCE 300 or 400 spectrometer (Bruker). Chemical shifts (δ) are reported in parts per million (ppm) with the residual solvent peak as an internal reference. High resolution mass spectrometry (HRMS) was carried out with time-of-flight electrospray ionization (ESI-TOF) using a UPLC H-Class/XEVO G2-XS (Waters Acquity). All starting materials and solvents were purchased from commercial suppliers and used without further purification. LiBOB was obtained from BLDpharm (99.94%). Eluent solvents used for flash column chromatography were purified by distillation prior to use. Reported R_F values were obtained using thin-layer chromatography (Macherey-Nagel ALUGRAM® SIL G UV_{254} TLC aluminum sheets silica gel 60 with fluorescent indicator, mean pore size 60 Å, 5-17 µm particle size, 0.20 mm layer thickness).

2. (Electro)chemical properties of LiBOB and reference salts

2.1 Chemical stability

Measurements of the chemical stability of LiBOB (see Figure 1) were carried out in CH_3CN, CH_3CN/H_2O (9:1, v/v), and CH_3OH using ^{11}B NMR spectroscopy at room temperature. A small amount of the respective deuterated solvent (CD_3CN or CD_3OD) was added to the solution prior to the measurement.

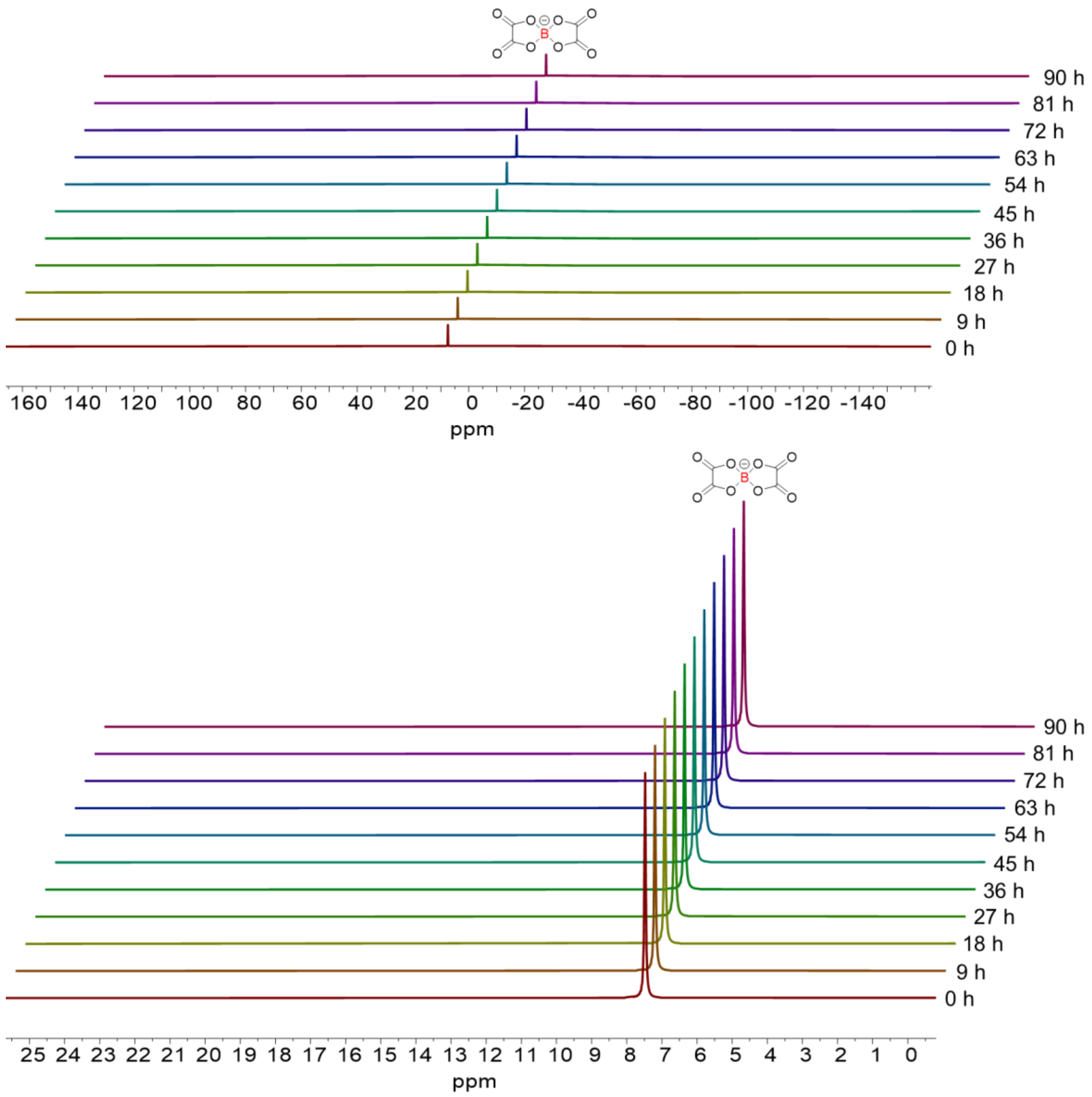

Figure S1. Time-dependent ^{11}B NMR analysis of a 0.2 M solution of LiBOB in CH_3CN. Top: Full spectra. Bottom: Enlarged sections illustrating the stability of BOB.

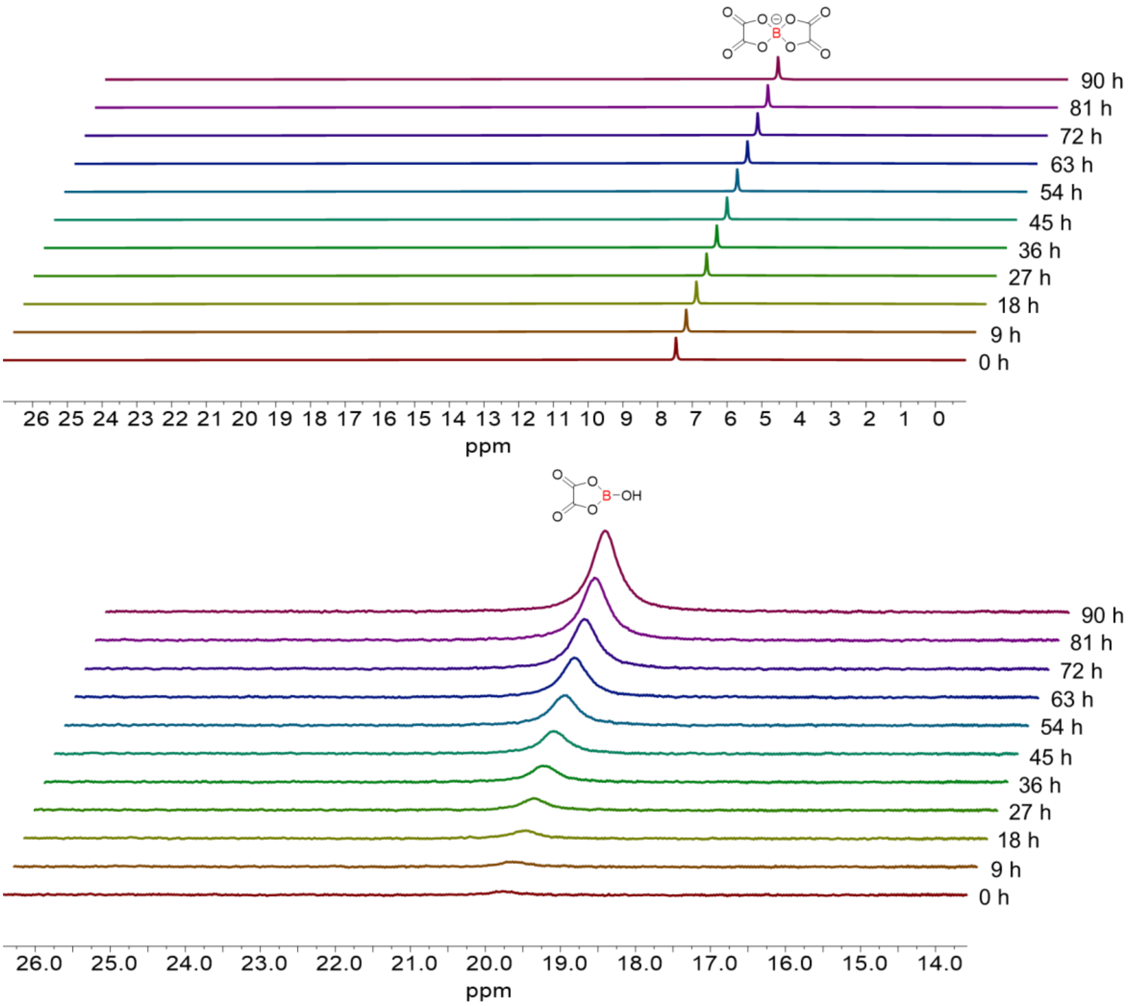

Figure S2. Time-dependent ^{11}B NMR analysis of a 0.2 M solution of LiBOB in CH_3CN/H_2O (9:1). Top: Full spectra. Bottom: Enlarged sections illustrating BOB degradation.

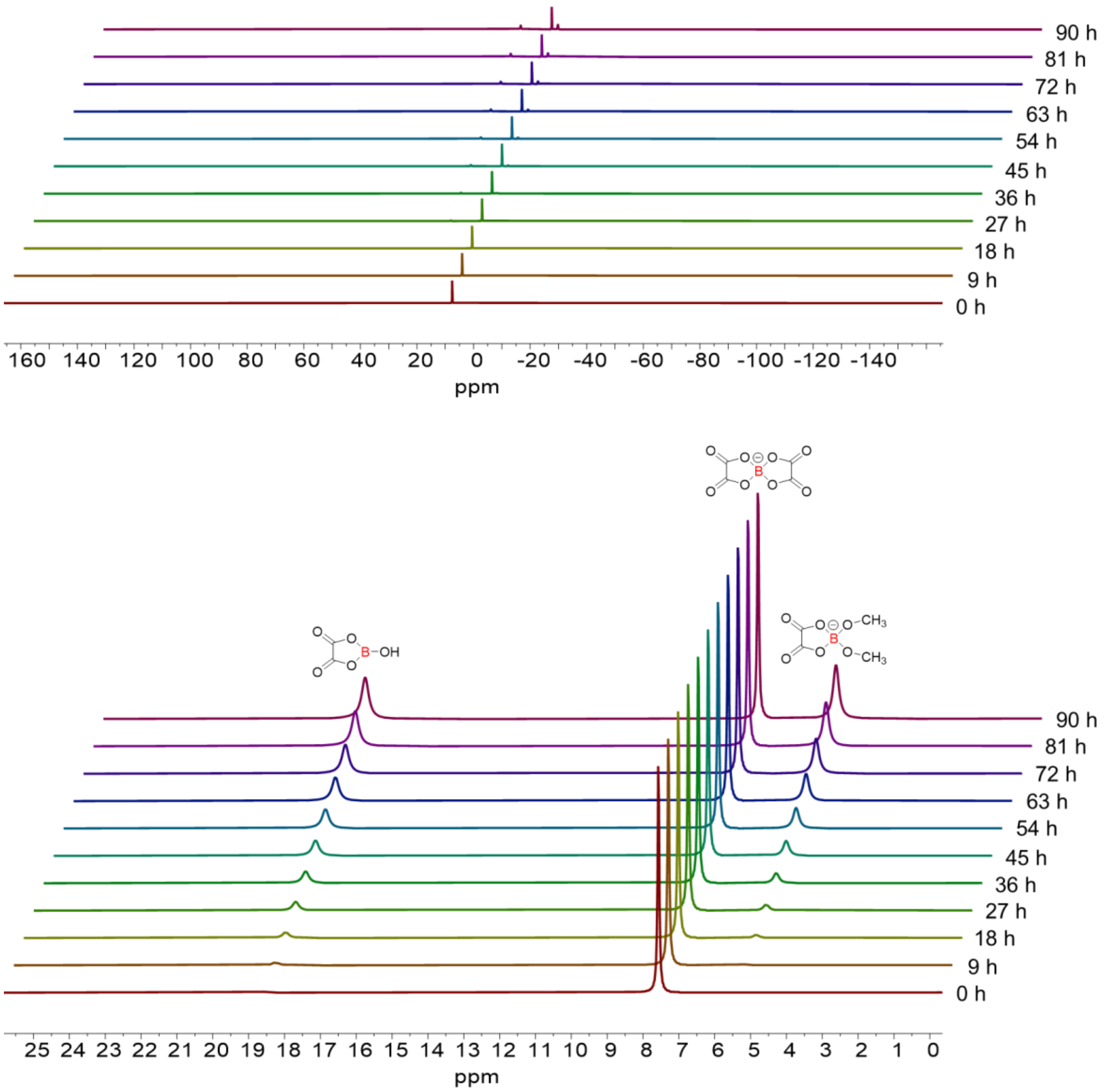

Figure S3. Time-dependent ^{11}B NMR analysis of a 0.2 M solution of LiBOB in CH_3OH. Top: Full spectra. Bottom: Enlarged sections illustrating BOB degradation.

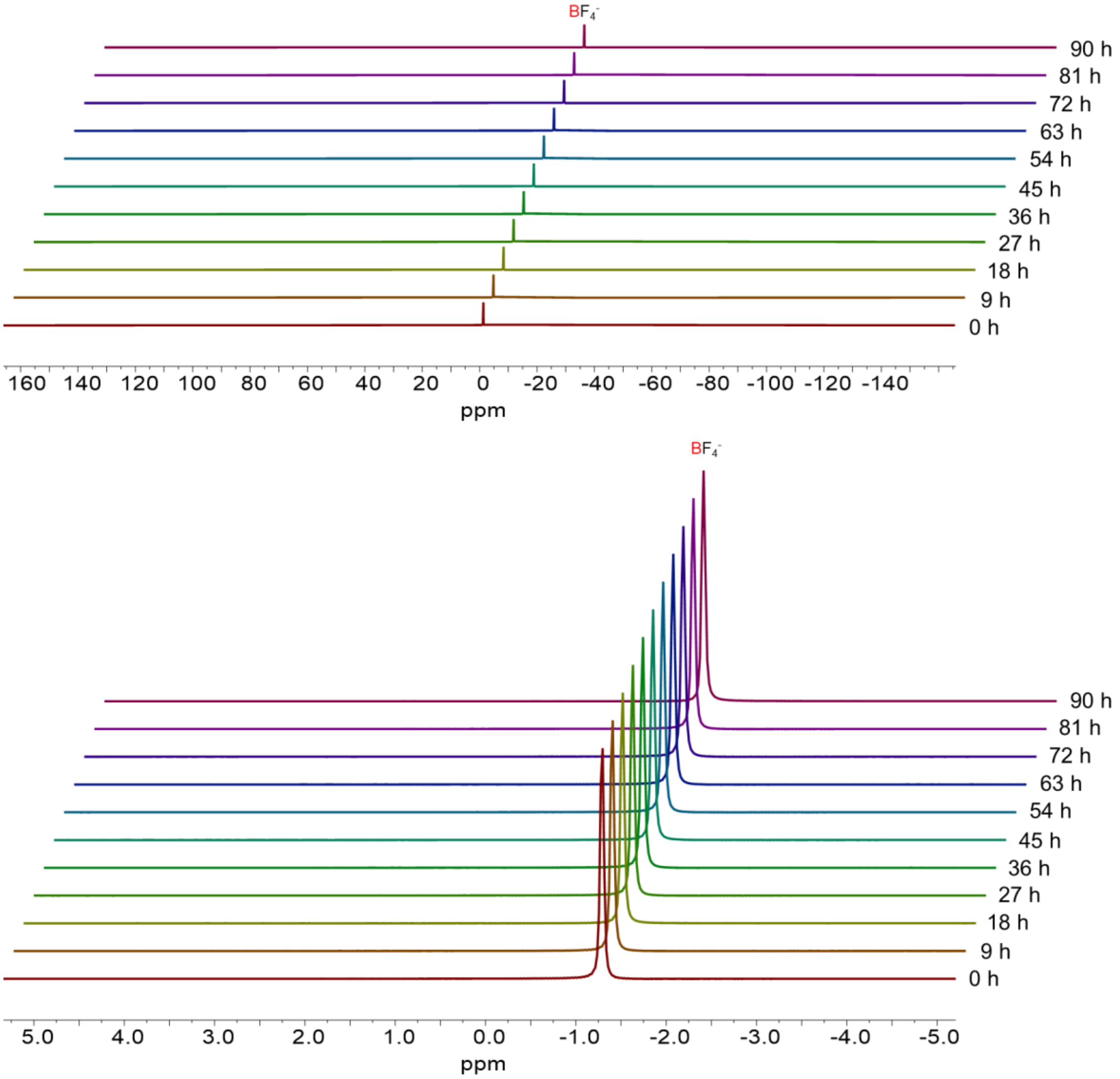

Figure S4. Time-dependent ^{11}B NMR analysis of a 0.2 M solution of $LiBF_4$ in CH_3CN. Top: Full spectra. Bottom: Enlarged sections illustrating the stability of the BF_4^- species.

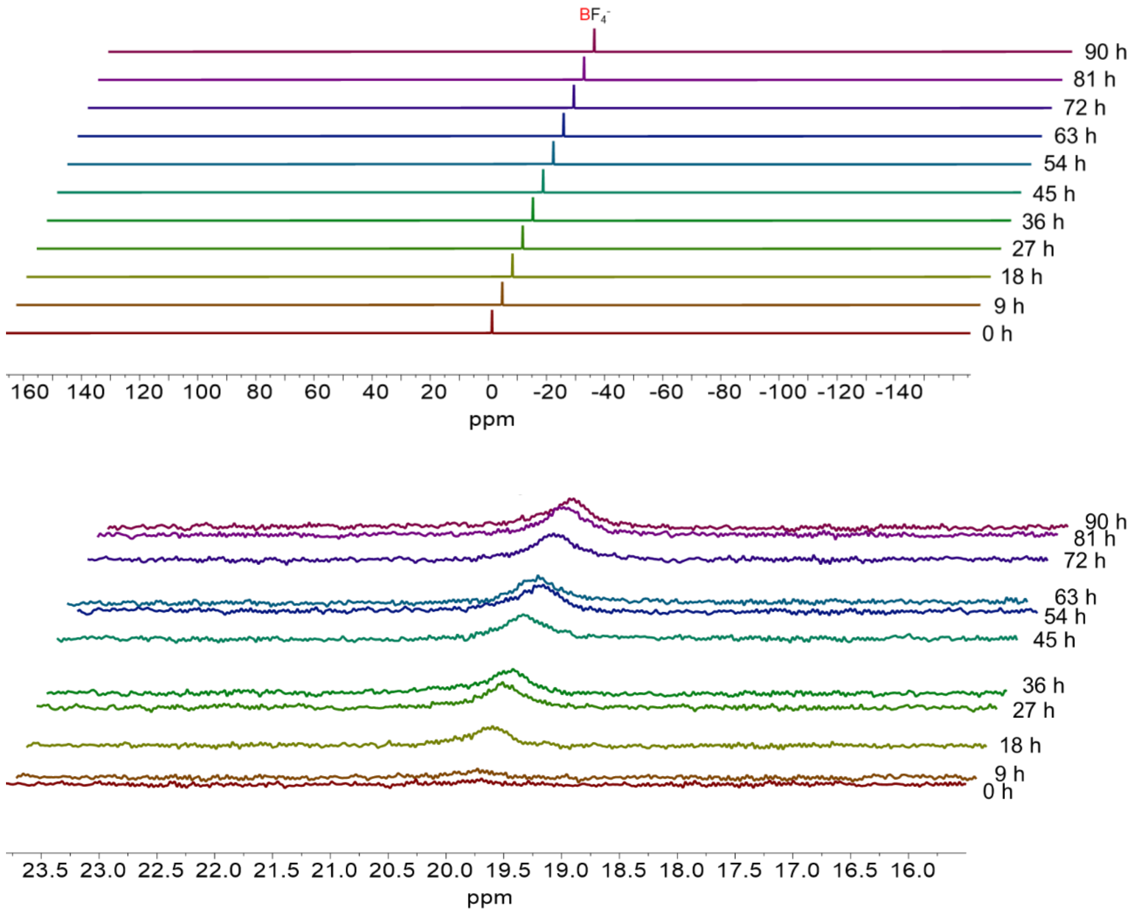

Figure S5. Time-dependent ^{11}B NMR analysis of a 0.2 M solution of $LiBF_4$ in CH_3CN/H_2O (9:1). Top: Full spectra. Bottom: Enlarged sections illustrating formation of a degradation product.

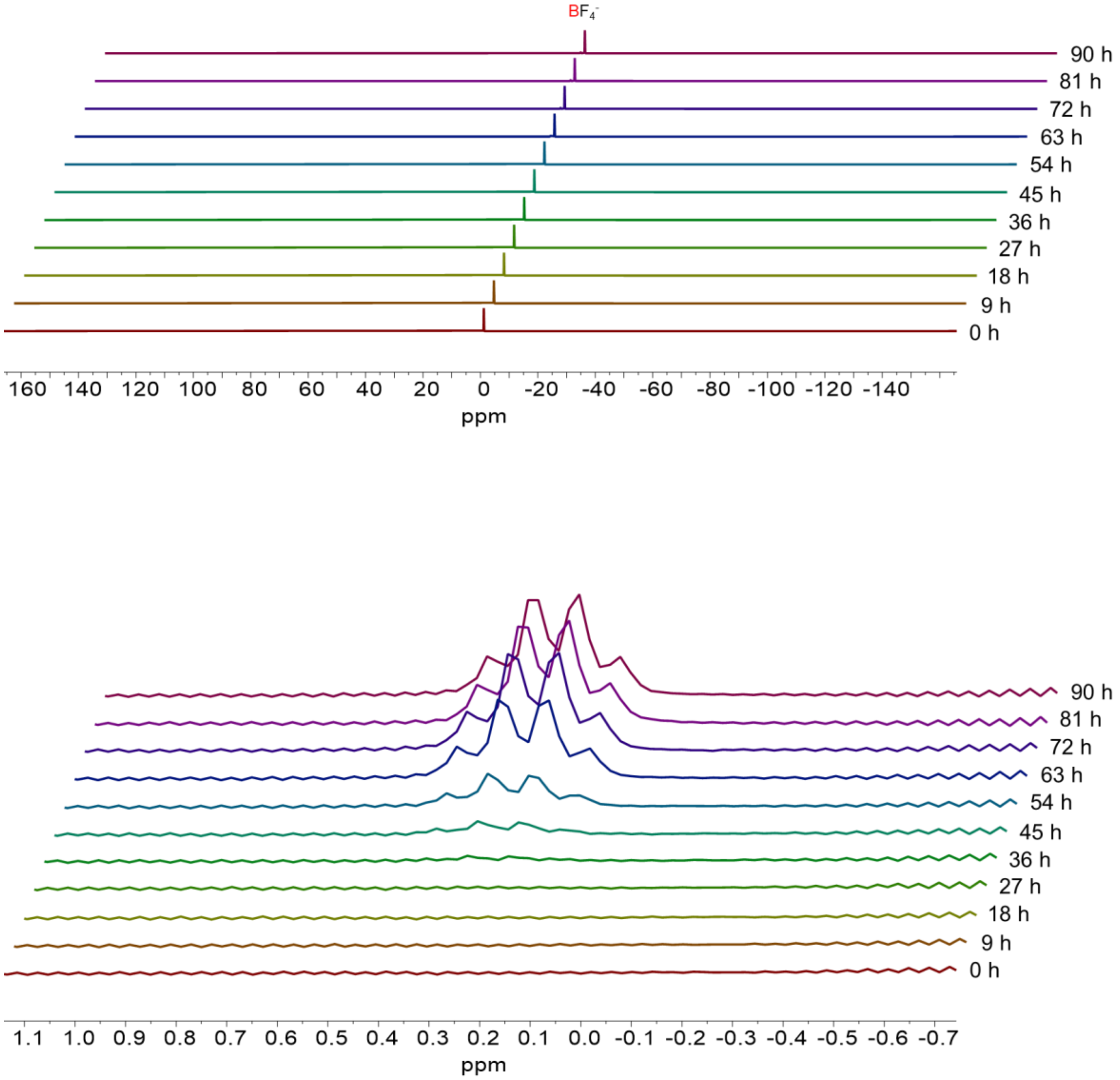

Figure S6. Time-dependent ^{11}B NMR analysis of a 0.2 M solution of $LiBF_4$ in CH_3OH. Top: Full spectra. Bottom: Enlarged sections illustrating formation of a degradation product.

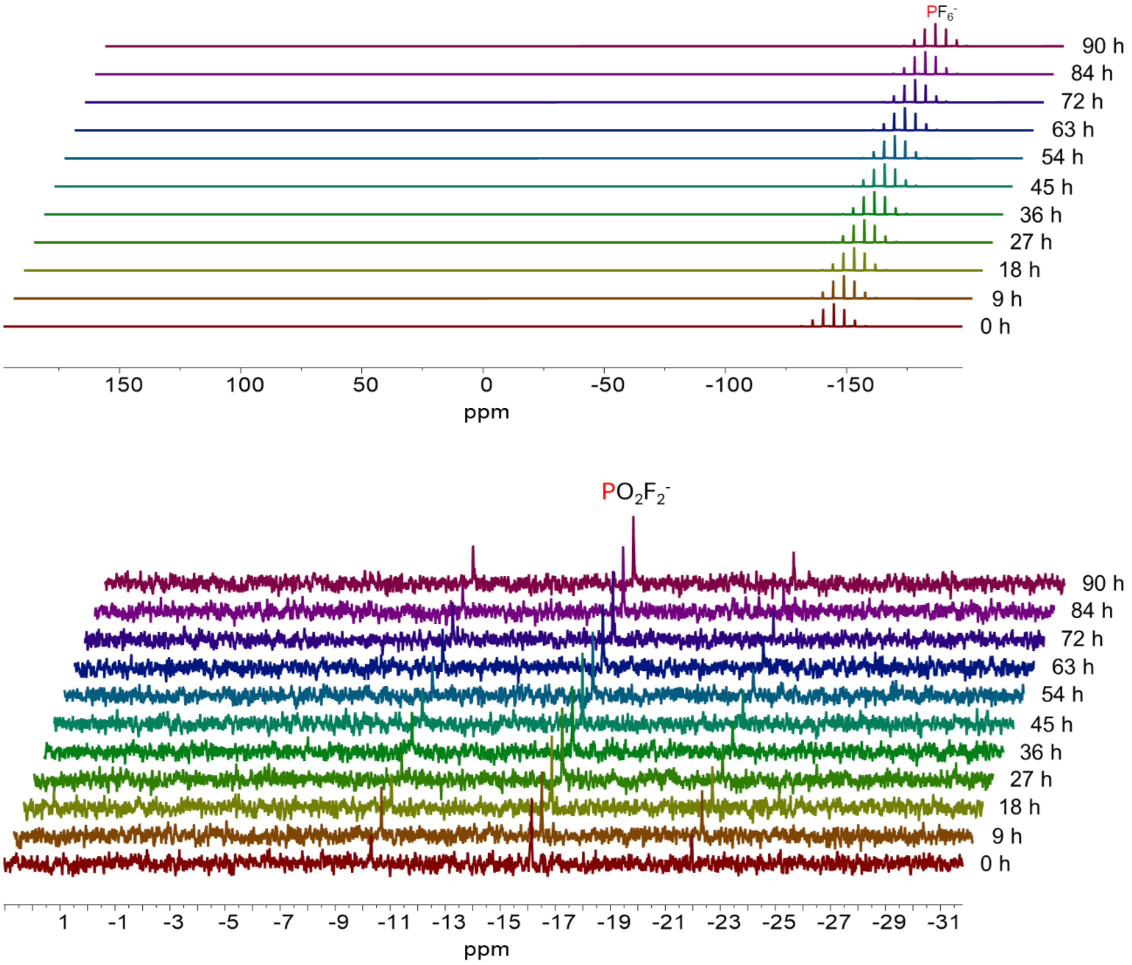

Figure S7. Time-dependent ^{31}P NMR analysis of a 0.2 M solution of $LiPF_6$ in CH_3CN. Top: Full spectra. Bottom: Enlarged sections illustrating the presence of a degradation product.

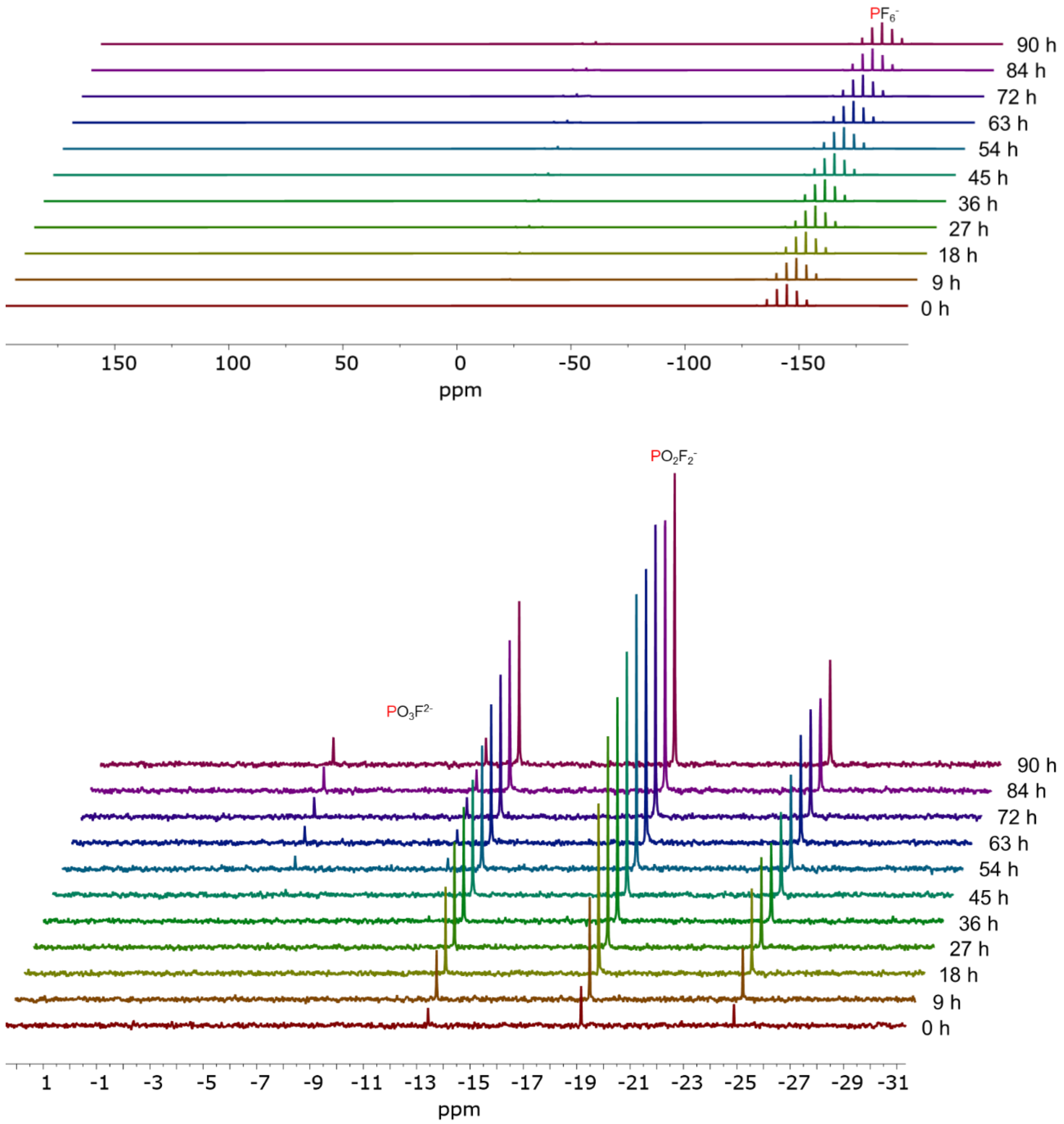

Figure S8. Time-dependent ^{31}P NMR analysis of a 0.2 M solution of $LiPF_6$ in CH_3CN/H_2O (9:1). Top: Full spectra. Bottom: Enlarged sections illustrating formation of degradation products.

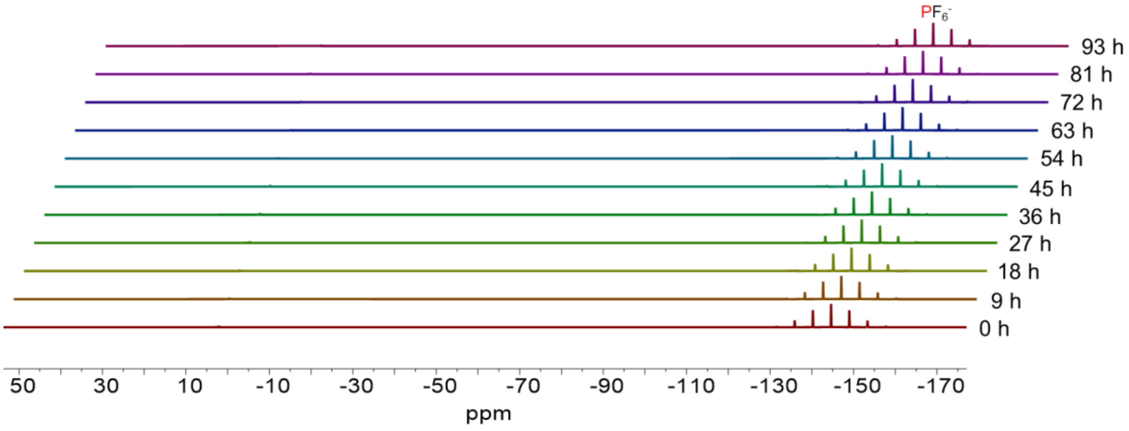

(a)

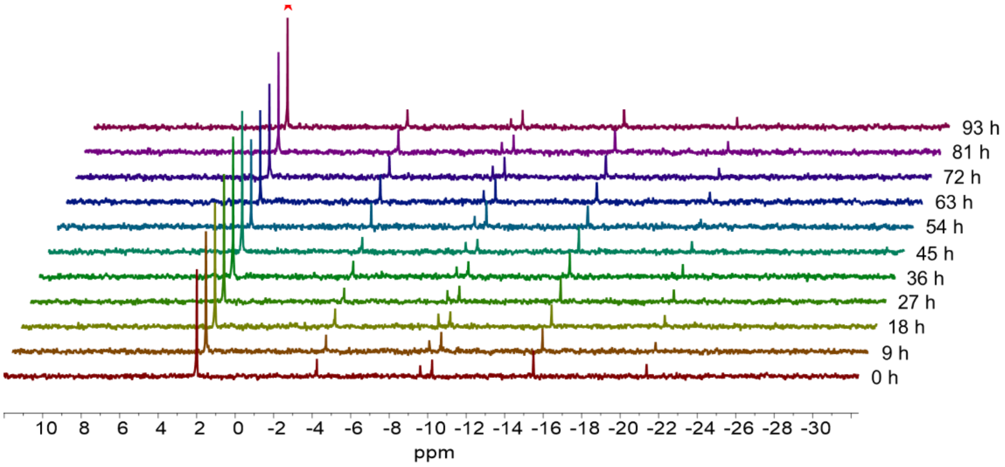

Figure S9. Time-dependent ^{31}P NMR analysis of a 0.2 M solution of $LiPF_6$ in CH_3OH. Top: Full spectra. Bottom: Enlarged sections illustrating the presence of degradation products.

2.2 Electrochemical stability

The electrochemical stabilities of solutions of LiBOB and reference salts were determined by cyclic voltammetry in a custom-made three-electrode cell using a PGSTAT 302N (Metrohm Autolab). A glassy carbon disk (diameter: 1.6 mm) served as the working electrode and a platinum wire as the counter electrode. Solutions of the respective salt (c = 0.1 M) in either CH_3CN or PC-DMC (4:1, *w*/*w*) were used as the electrolyte. The glassy carbon disk was polished using polishing alumina suspension (0.05 μm) prior to each experiment. As reference, a Ag/$AgNO_3$ electrode (silver wire in 0.1 M Bu_4NClO_4/ CH_3CN solution; $c(AgNO_3)$ = 0.01 M; E^0 = -87 mV vs. ferrocene/ferrocenium couple)[1] was used, and this compartment was separated from the rest of the cell with a Vycor frit. The analyte[2] solutions were purged with argon for at least 5 minutes prior to the experiment. Anodic and cathodic scans were recorded separately, each at 10 mV s^{-1}. The stability limit was determined by reading out the potential required for reaching a current density of $|0.1\ mA\ cm^{-2}|$. The reported values are the averages from three measurements.

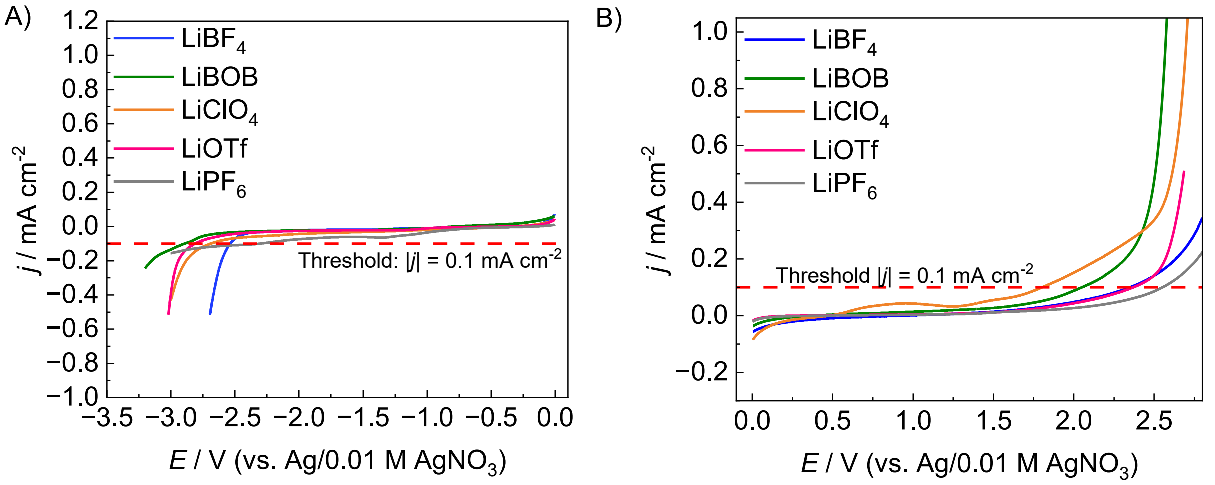

Figure S10. Voltammetric analysis of 0.1 M supporting electrolyte solutions in acetonitrile. A) Linear sweeps in the reductive regime. B) Linear sweeps in the oxidative regime. The shown LSVs are the forward scans of the third cycles taken from CV measurements recorded under the conditions described above.

[1] V. V. Pavlishchuk, A. W. Addison, *Inorg. Chim Acta* **2000**, *298*, 97.
[2] Specifications of lithium salts used: $LiBF_4$ (99.99% trace metals basis, Sigma-Aldrich), LiBOB (99.94%, BLDpharm), $LiClO_4$ (99+% for analysis, Thermo Fisher), LiOTf (99.995% trace metals basis, Sigma-Aldrich), $LiPF_6$ (battery grade ≥99.99% trace metals basis, Sigma-Aldrich).

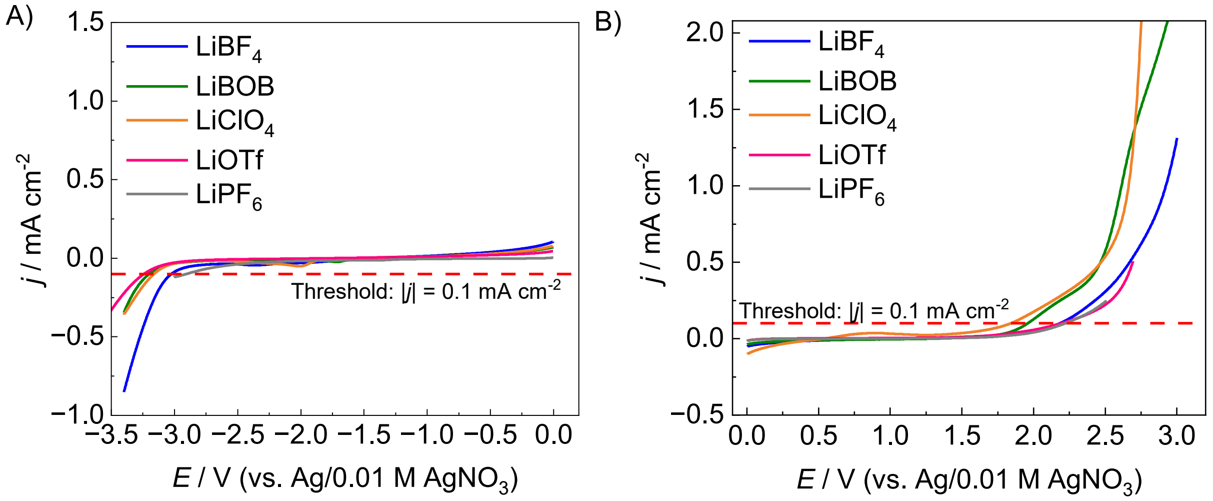

Figure S11. Voltammetric analysis of 0.1 M supporting electrolytes in PC/DMC (4:1 *w*/*w*). A) Linear sweeps in the reductive regime. B) Linear sweeps in the oxidative regime. The shown LSVs are the forward scans of the third cycles taken from CV measurements recorded under the conditions described above.

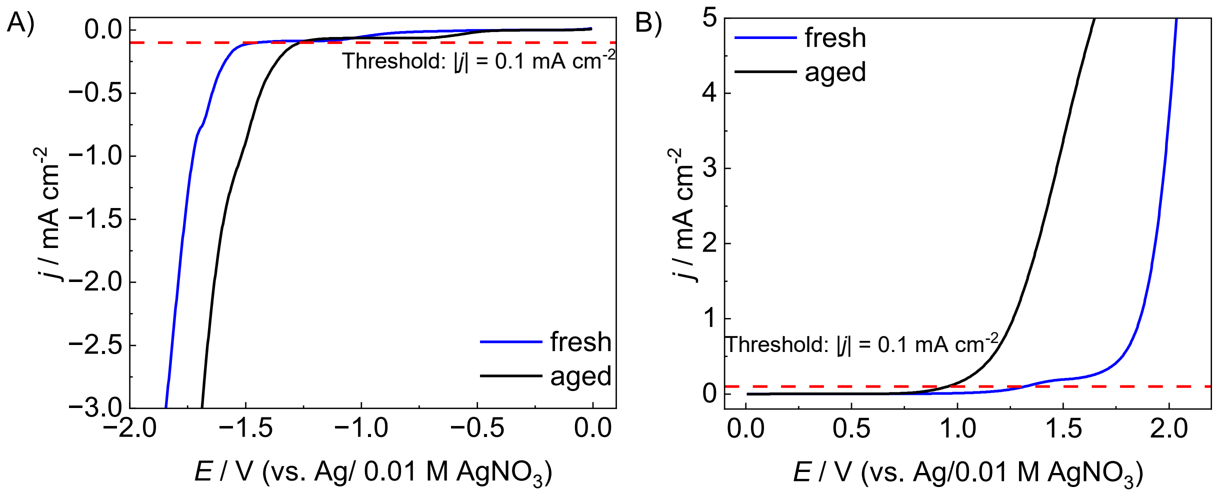

Figure S12. Voltammetric analysis of a fresh (blue line) and aged (black line, 90 h at room temperature) 0.1 M solution of LiBOB in MeOH. A) Linear sweeps in the reductive regime. B) Linear sweeps in the oxidative regime. The shown LSVs are the forward scans of the third cycles taken from CV measurements recorded under the conditions described above.

Table S1. Anodic and cathodic stability limits (E_{red} and E_{ox}) determined at 10 mV s^{-1} using 0.1 M solutions of the respective salt (see Figure S10 and Figure S11).

**Salt**	**Solvent**	**E_{red} [V]**	**E_{ox} [V]**
LiOTf	PC-DMC (4:1)	-3.19±0.05	2.187±0.015
$LiBF_4$	PC-DMC (4:1)	-3.021±0.004	2.14±0.07
$LiPF_6$	PC-DMC (4:1)	-2.94±0.04	2.231±0.010
$LiClO_4$	PC-DMC (4:1)	-3.155±0.009	1.865±0.023
LiBOB	PC-DMC (4:1)	-3.15±0.05	1.938±0.029
LiOTf	CH_3CN	-2.84±0.04	2.372±0.006
$LiBF_4$	CH_3CN	-2.533±0.011	2.28±0.11
$LiPF_6$	CH_3CN	-2.36±0.11	2.546±0.007
$LiClO_4$	CH_3CN	-2.64±0.07	1.76±0.04
LiBOB	CH_3CN	-2.913±0.026	1.98±0.09
LiBOB (freshly prepared)	CH_3OH	-1.42±0.04	1.309±0.022
LiBOB (aged, 96 h)	CH_3OH	-1.277±0.029	0.953±0.003

2.3 Ionic conductivity

Conductivity values were measured using a Mettler Toledo Seven Compact Duo S213 device in a custom-made glass cell at 25 °C for at least three times. Prior to each series of measurements, the device was calibrated using a commercially available KCl conductivity standard solution (ROTH, ROTI®CALIPURE 12880 µS/cm ± 1% @25 °C)

Table S2. Conductivity values determined for LiBOB and reference salts at 25 °C in acetonitrile for various concentrations.

c [mol L^{-1}]	lithium salt	*σ* [mS cm^{-1}]
0.05	$LiClO_4$	5.38±0.04
	$LiBF_4$	4.391±0.018
	$LiPF_6$	4.25±0.04
	LiBOB	4.32±0.09
	LiOTf	3.32±0.06
0.10	$LiClO_4$	9.16±0.05
	$LiBF_4$	7.0±0.4
	$LiPF_6$	8.17±0.07
	LiBOB	8.10±0.11
	LiOTf	4.47±0.07
0.15	$LiClO_4$	12.05±0.08
	$LiBF_4$	8.57±0.14
	$LiPF_6$	11.34±0.16
	LiBOB	11.31±0.23
	LiOTf	5.45±0.04
0.20	$LiClO_4$	14.73±0.11
	$LiBF_4$	9.79±0.08
	$LiPF_6$	13.62±0.29
	LiBOB	14.01±0.11
	LiOTf	6.31±0.11
0.25	$LiClO_4$	17.02±0.10
	$LiBF_4$	12.4±0.9
	$LiPF_6$	15.52±0.22
	LiBOB	17.02±0.05
	LiOTf	6.78±0.05
0.30	$LiClO_4$	19.21±0.16
	$LiBF_4$	13.6±0.8
	$LiPF_6$	17.95±0.11
	LiBOB	19.133±0.029
	LiOTf	7.20±0.05
0.40	$LiClO_4$	22.3±0.5
	$LiBF_4$	13.78±0.09
	$LiPF_6$	19.53±0.16
	LiBOB	20.4±0.7
	LiOTf	8.16±0.20
0.50	$LiClO_4$	25.73±0.16
	$LiBF_4$	16.5±0.4
	$LiPF_6$	21.10±0.12
	LiBOB	26.37±0.06
	LiOTf	8.52±0.12

Table S3. Conductivity values determined for LiBOB and reference salts at 25 °C in PC/DMC (4:1, *w*/*w*) for various concentrations.

c [mol L^{-1}]	lithium salt	σ [mS cm^{-1}]
0.05	$LiClO_4$	1.245±0.016
	$LiBF_4$	1.138±0.010
	$LiPF_6$	1.05±0.05
	LiBOB	1.008±0.006
	LiOTf	0.864±0.011
0.10	$LiClO_4$	2.318±0.020
	$LiBF_4$	1.899±0.021
	$LiPF_6$	1.987±0.006
	LiBOB	1.938±0.025
	LiOTf	1.38±0.07
0.15	$LiClO_4$	3.06±0.04
	$LiBF_4$	2.451±0.004
	$LiPF_6$	2.532±0.026
	LiBOB	2.576±0.021
	LiOTf	1.68±0.04
0.20	$LiClO_4$	3.74±0.05
	$LiBF_4$	2.94±0.06
	$LiPF_6$	3.10±0.11
	LiBOB	3.02±0.06
	LiOTf	1.944±0.028
0.25	$LiClO_4$	4.33±0.07
	$LiBF_4$	3.237±0.022
	$LiPF_6$	3.625±0.020
	LiBOB	3.543±0.017
	LiOTf	2.149±0.016
0.30	$LiClO_4$	4.79±0.08
	$LiBF_4$	3.574±0.029
	$LiPF_6$	4.15±0.04
	LiBOB	3.914±0.029
	LiOTf	2.264±0.025
0.40	$LiClO_4$	5.47±0.07
	$LiBF_4$	3.942±0.030
	$LiPF_6$	4.578±0.014
	LiBOB	4.39±0.07
	LiOTf	2.393±0.025
0.50	$LiClO_4$	6.06±0.11
	$LiBF_4$	4.302±0.029
	$LiPF_6$	5.119±0.018
	LiBOB	4.866±0.029
	LiOTf	2.52±0.06

3. Preparative-scale electrolysis

3.1 Electrosynthesis of diaryliodonium compounds

General procedure: The electrosynthesis of bis(4-bromophenyl)iodonium bis(oxalato)borate was carried out according to a procedure adapted from the literature.[3] All electrolyses were performed at room temperature in a custom-made H-type divided glass cell with a G4 frit as separator. A Rohde & Schwarz HMP 4040 galvanostat served as the power source. The anolyte solution was prepared by dissolving *p*-bromoiodobenzene (**5**, 1.0 mmol, 1.0 equiv., 0.2 M), bromobenzene, and lithium bis(oxalato)borate in acetonitrile (5 mL). The catholyte solution consisted of lithium bis(oxalato)borate in acetonitrile (5 mL, same salt concentration as in the anolyte). After complete dissolution of all compounds, both solutions were carefully added to their respective half-cell compartment *via* syringes at the same time. A glassy carbon plate (thickness: 3 mm, width: 10 mm, immersion depth: 1 cm, SIGRADUR G, HTW GmbH, Germany) was used as the cathode and a platinum sheet as the anode (width: 10 mm, immersion depth: 1 cm). The distance between both electrodes was 3.5 cm. Reactions were carried out under atmospheric conditions at room temperature at j = 5 mA cm^{-2}.

Quantification of **7** was achieved by ^{1}H NMR spectroscopy using an internal standard. For this purpose, a defined amount of mesitylene was added to the anolyte solution. An aliquot (10% of the electrolyte solution) was then diluted with CD_3CN and subjected to ^{1}H NMR analysis. The stability of the BOB anion was assessed by ^{11}B NMR spectroscopy using the same analyte solution (see Figure S13). No degradation could be observed even after passing 4.0 F per mole **5**.

For isolation of **7**, the remaining 90% of the electrolyte was concentrated and subjected to normal phase flash column chromatography using dichloromethane/acetonitrile (3:1) as eluent. The product was obtained as colorless powder (473 mg, 84% isolated yield, taking into account the aliquot removed for product quantification by ^{1}H NMR spectroscopy). R_f = 0.44 (dichloromethane/acetonitrile 3:1). ^{1}H NMR (300 MHz, CD_3CN) δ 8.0 (d, J = 8.8 Hz, 4H), 7.7 (d, J = 8.8 Hz, 4H). ^{11}B NMR (96 MHz, CD_3CN) δ 7.46 (s). ^{13}C{^{1}H} NMR (75 MHz, CD_3CN) δ 159.7, 138.1, 136.6, 128.9, 112.4. HRMS (ESI-TOF) m/z: calcd. for $C_{12}H_8Br_2I^+$ 436.8033; found 436.8045. Calcd. for $C_4O_8{}^{11}B$ 186.9692; found 186.9690. Calcd for $C_4O_8{}^{10}B$ 185.9728; found 185.9725.

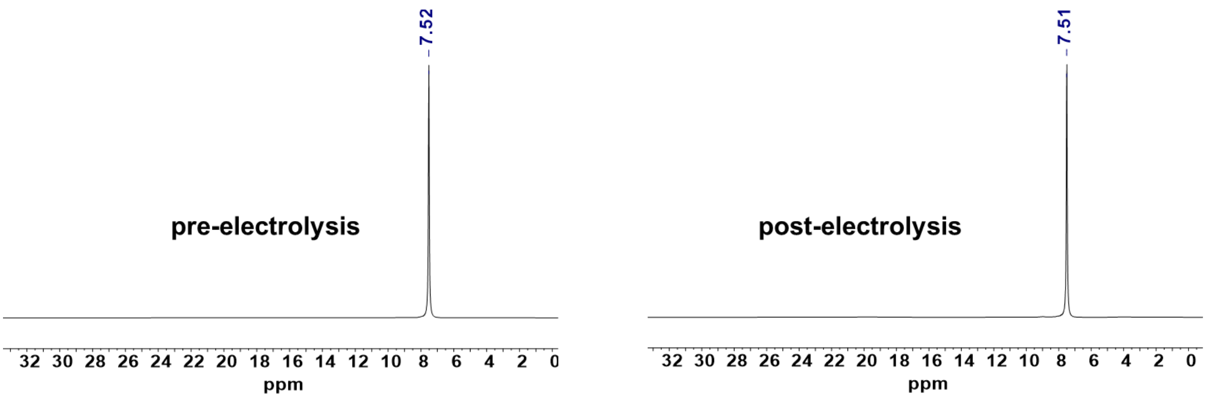

Figure S13. Monitoring the stability of the BOB anion during electrochemical synthesis of diaryl iodonium salt **7**. ^{11}B NMR spectra of a freshly prepared reaction mixture (left) and after passing 4 F per mole **5** (right, for electrolysis conditions, see general procedure). Electrolyte aliquots were diluted with CD_3CN for NMR analysis.

---

[3] Scherkus, A.; Gudkova, A.; Čada, J.; Müller, B. H.; Bystron, T.; Francke, R. Low-Cost, Safe, and Anion-Flexible Method for the Electrosynthesis of Diaryliodonium Salts. *J. Org. Chem.* **2024**, 89, 19, 14129-14134.

3.2 TEMPO-mediated oxidation of alcohols

General procedure: The TEMPO-mediated oxidation of alcohols was achieved by adapting a procedure from the literature.[4] All electrolyses were carried out in a custom-made undivided glass cell. A Rohde & Schwarz HMP 4040 galvanostat served as the power source. The electrolyte was prepared by adding the corresponding alcohol (1.0 mmol, 0.067 M), TEMPO (15.6 mg, 0.1 mmol, 0.1 equiv., 67 mM) and LiBOB (290.7 mg, 1.5 mmol, 1.5 equiv., 0.1 M) to the electrolysis cell. Afterwards, 15 mL acetonitrile were added, and the mixture was stirred until complete dissolution of all solids was achieved, followed by addition of *N*-methylimidazole (359 µL, 4.5 mmol, 4.5 equiv., 0.3 M). Graphite plates served as electrodes (thickness: 3 mm, width: 10 mm, immersion depth: 2 cm). The distance between the electrodes was 1.3 cm. Reactions were carried out under atmospheric conditions at room temperature applying 6 mA cm^{-2} and three charge equivalents per mole alcohol (Q = 3.0 F).

Quantification of **9** was achieved by ^{1}H NMR spectroscopy using an internal standard. For this purpose, a defined amount of 1,3,5-trimethoxybenzene was added to the electrolyte solution. An aliquot (10% of the electrolyte solution) was then diluted with CD_3CN and subjected to ^{1}H NMR analysis. The stability of the BOB anion was assessed by ^{11}B NMR spectroscopy using the same analyte solution (see Figure S14). No degradation could be observed even after passing 3.0 F per mole **8**.

Table S4. Optimization of the LiBOB concentration under the conditions described in the general procedure (yields determined by ^{1}H NMR spectroscopy using 1,3,5-trimethoxybenzene as internal standard).

Entry	c_{LiBOB} [mol L^{-1}]	**9a** [%]
1	0.05	82
2	0.10	86
3	0.20	88

Due to their volatility, compounds **9c**, **9d**, and **9e** were only quantified by ^{1}H NMR spectroscopy and not isolated. For isolation of **9a** and **9b**, the remaining 90% of the electrolyte was concentrated under reduced pressure and subjected to flash column chromatography using *n*-pentane/ethyl acetate 4:1 as eluent.

[1,1'-Biphenyl]-4-carbaldehyde (**9a**) was obtained as a colorless solid (139 mg, 85% isolated yield, taking into account the aliquot removed for product quantification by ^{1}H NMR spectroscopy). R_f = 0.40 (*n*-pentane/ethyl acetate 4:1). ^{1}H NMR (300 MHz, CD_2Cl_2) δ 10.1 (s, 1H), 8.0 (d, J = 8.6 Hz, 2H), 7.8 (d, J = 8.1 Hz, 2H), 7.7 – 7.6 (m, 2H), 7.5 – 7.4 (m, 3H). $^{13}C\{^{1}H\}$ NMR (75 MHz, CD_2Cl_2) δ 191.8, 147.0, 139.7, 135.4, 130.1, 129.0, 128.5, 127.6, 127.3.

2-Naphthaldehyde (**9b**) was obtained as a colorless solid (108 mg, 77% isolated yield, taking into account the aliquot removed for product quantification by ^{1}H NMR spectroscopy). R_f = 0.57 (*n*-pentane/ethyl acetate 4:1). ^{1}H NMR (300 MHz, CD_2Cl_2) δ 10.2 (s, 1H), 8.4 (m, 1H), 8.1 – 8.0 (m, 1H), 8.0 – 7.9 (m, 3H), 7.7 – 7.6 (m, 2H). $^{13}C\{^{1}H\}$ NMR (75 MHz, CD_2Cl_2) δ 192.1, 136.4, 134.4, 134.2, 132.7, 129.5, 129.1, 129.0, 128.0, 127.1, 122.6.

[4] Prudlik, A.; Matei, A.; Scherkus, A; Bardagi, J. I.; Beil, S. B.; Francke, R. On the use of propylene carbonate and dimethyl carbonate as green solvents in organic electrosynthesis. *Green Chem.* **2025**, 27, 4280-4288.

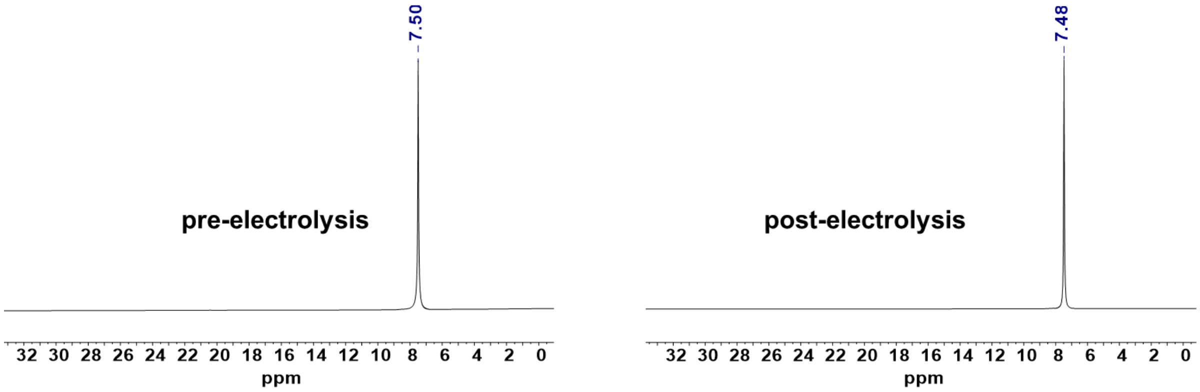

Figure S14. Monitoring the stability of the BOB anion during TEMPO-catalyzed alcohol oxidation. Exemplary ^{11}B NMR spectra of a freshly prepared reaction mixture (left) and after passing 3 F per mole alcohol (right, for electrolysis conditions, see general procedure). Aliquots of the electrolyte solution were diluted with CD_3CN for NMR analysis.

3.3 Cathodic reduction of benzophenone

Synthesis of Et_4NBOB: Tetraethylammonium bis(oxalato)borate was synthesized by adapting a procedure reported in the literature.[5] Lithium bis(oxalato)borate (15.0 mmol, 2.91 g) and tetraethylammonium chloride (15.0 mmol, 2.49 g) were dissolved in acetonitrile (15 mL) and stirred overnight. The precipitate was filtered off, followed by evaporation of the solvent from the filtrate under reduced pressure. The remaining solid was dissolved in 30 mL dichloromethane and transferred into a separatory funnel. After adding 5 mL of water and thorough mixing, the organic layer was separated and dried over $MgSO_4$. The solvent was removed under reduced pressure and the product was further dried at 50 °C under vacuum. Et_4NBOB was obtained as a colorless solid (4.49 g, 14.2 mmol, 94% yield). ^{1}H NMR (400 MHz, CD_3CN) δ 3.2 (q, J = 7.3 Hz, 8H), 1.2 (t, J = 7.3 Hz, 12H). ^{11}B NMR (128 MHz, CD_3CN) δ 7.5. $^{13}C\{^{1}H\}$ NMR (101 MHz, CD_3CN) δ 159.7, 53.0, 7.6.

General electrolysis protocol: The direct cathodic reduction of benzophenone (**10**) was conducted by adapting a procedure reported in the literature.[4] Oxygen was removed from the solvents by purging with argon for 15 min. All electrolyses were carried out in a custom-made undivided batch-type glass cell with a three-electrode arrangement. As reference, a Ag/$AgNO_3$ electrode (silver wire in 0.1 M Bu_4NClO_4/CH_3CN solution; $c(AgNO_3)$ = 0.01 M; E^0 = -87 mV vs. ferrocene/ferrocenium couple)[6] was used, and this compartment was separated from the rest of the cell with a Vycor frit. The electrolyte was prepared by adding benzophenone (182 mg, 1.0 mmol, 0.067 M, if not stated otherwise), DABCO (3.0 equiv.), 100 µL of a proton donor (MeOH or H_2O, see Table S5), and Et_4NBOB (for concentrations, see Table S5) to the electrolysis cell. Afterwards, 15 mL of solvent were added, and the mixture was stirred until achieving dissolution of all compounds. Glassy carbon plates (SIGRADUR G, HTW GmbH, Germany) served as working electrode and counter electrode, respectively. The potential was maintained −2.2 V vs. Ag/0.01 M $AgNO_3$ throughout electrolysis and the cell kept under an argon atmosphere.

Quantification of **11** was achieved by ^{1}H NMR spectroscopy using an internal standard. For this purpose, a defined amount of 1,3,5-trimethoxybenzene was added to the electrolyte solution. An aliquot (10% of the electrolyte solution) was then diluted with CD_3CN and subjected to ^{1}H NMR analysis. The stability of the BOB anion was assessed by ^{11}B NMR spectroscopy using the same analyte solution (see Figure S15). Already in the freshly prepared solution containing Et_4NBOB, degradation product **3** is formed in small amounts, as indicated by the signal at 5.0 ppm. In the spectrum of the post-electrolysis solution, the BOB parent signal at 7.4 ppm disappeared completely and only the signals assigned to decomposition products **3** and **4** were detected.[7]

[5] Nguyen, H. V. T.; Lee, S.; Kwak, K.; Lee, K.-K. Bis(oxalate)borate-containing electrolytes for high voltage electric double-layer capacitors: A comparative study. *Electrochim. Acta* **2019**, 134649.
[6] V. V. Pavlishchuk, A. W. Addison, *Inorg. Chim Acta* **2000**, *298*, 97.
[7] Yang, L.; Furczon, M. M.; Xiao, A.; Lucht, B. L.; Zhang, Z.; Abraham, D. P. Effect of impurities and moisture on lithium bisoxalatoborate (LiBOB) electrolyte performance in lithium-ion cells. *J. Power Sources* **2010**, 195, 6, 1698-1705.

Table S5. Optimization of the cathodic reduction of benzophenone (**10**) to diphenylmethanol (**11**) in an undivided cell at –2.2 V vs. Ag/$AgNO_3$. Diphenylmethane (**12**) occurs as a side product. Isolated yields are added in parentheses (entries 1, 2, 11, and 12).

Entry	Proton source [a]	Solvent	*C*(Et_4NBOB) [mol L^{-1}]	Charge equiv. [F]	**10** [%] [b]	**11** [%] [b]	**12** [%] [b]
1	CH_3OH	CH_3CN	0.075	4.0	0	81 (81)	11
2 [c]	CH_3OH	CH_3CN	0.075	4.0	0	91 (90)	0
3	CH_3OH	CH_3CN	0.025	4.0	66	21	11
4	CH_3OH	CH_3CN	0.010	4.0	93	5	2
5	CH_3OH	CH_3CN	0.075	3.0	22	68	7
6	H_2O	PC/DMC (4:1)	0.150	2.0	41	34	14
7	H_2O	PC/DMC (4:1)	0.150	3.0	13	54	20
8	CH_3OH	PC/DMC (4:1)	0.150	3.0	24	64	8
9	CH_3OH	PC/DMC (4:1)	0.150	4.0	0	78	13
10	CH_3OH	PC/DMC (4:1)	0.150	5.5	5	74	11
11	CH_3OH	PC/DMC (4:1)	0.075	4.0	0	87 (68)	4
12 [c]	CH_3OH	PC/DMC (4:1)	0.075	2.3 [e]	0	89 (69)	0

[a] 100 µL of proton donor added per millimole of **10**. [b] Yields determined by ^1H NMR spectroscopy using 1,3,5-trimethoxybenzene as internal standard. [c] Batch size: 5 mmol of **10**. [e] Current dropped to baseline after passing 2.3 F per mole **10**.

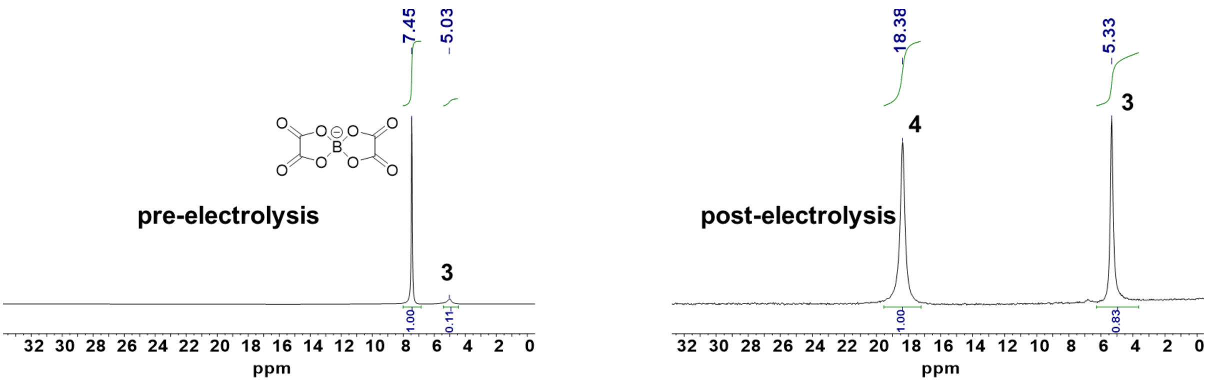

Figure S15. Monitoring the conversion of BOB during cathodic reduction of benzophenone using MeOH as a proton donor. Exemplary ^{11}B NMR spectra of a freshly prepared reaction mixture (left) and after passing 2 F per mole **10** (right, for electrolysis conditions, see general procedure). Electrolyte aliquots were diluted with CD_3CN for NMR analysis.

For isolation of diphenylmethanol (**11**), the solvent was removed from the electrolyte under reduced pressure. Afterwards, the residue was subjected to flash column chromatography using *n*-pentane/ethyl acetate 9:1 as eluent. **11** was obtained as a colorless solid (134 mg, 727 µmol, 81% isolated yield at 1 mmol scale; 747 mg, 405 µmol, 90% isolated yield at 5 mmol scale, each taking into account the aliquot removed for product quantification by ^1H NMR spectroscopy). ^1H NMR (300 MHz, CD_2Cl_2) δ 7.5 – 7.2 (m, 10H), 5.8 (s, 1H), 2.5 (s, 1H). ^{13}C NMR (75 MHz, CD_2Cl_2) δ 144.6, 128.8, 127.8, 126.8, 76.4.

4. NMR spectra

4.1 Analysis of isolated compounds

^{1}H NMR spectrum (300 MHz, CD_3CN) of bis(4-bromophenyl)iodonium bis(oxalato)borate (**7**).

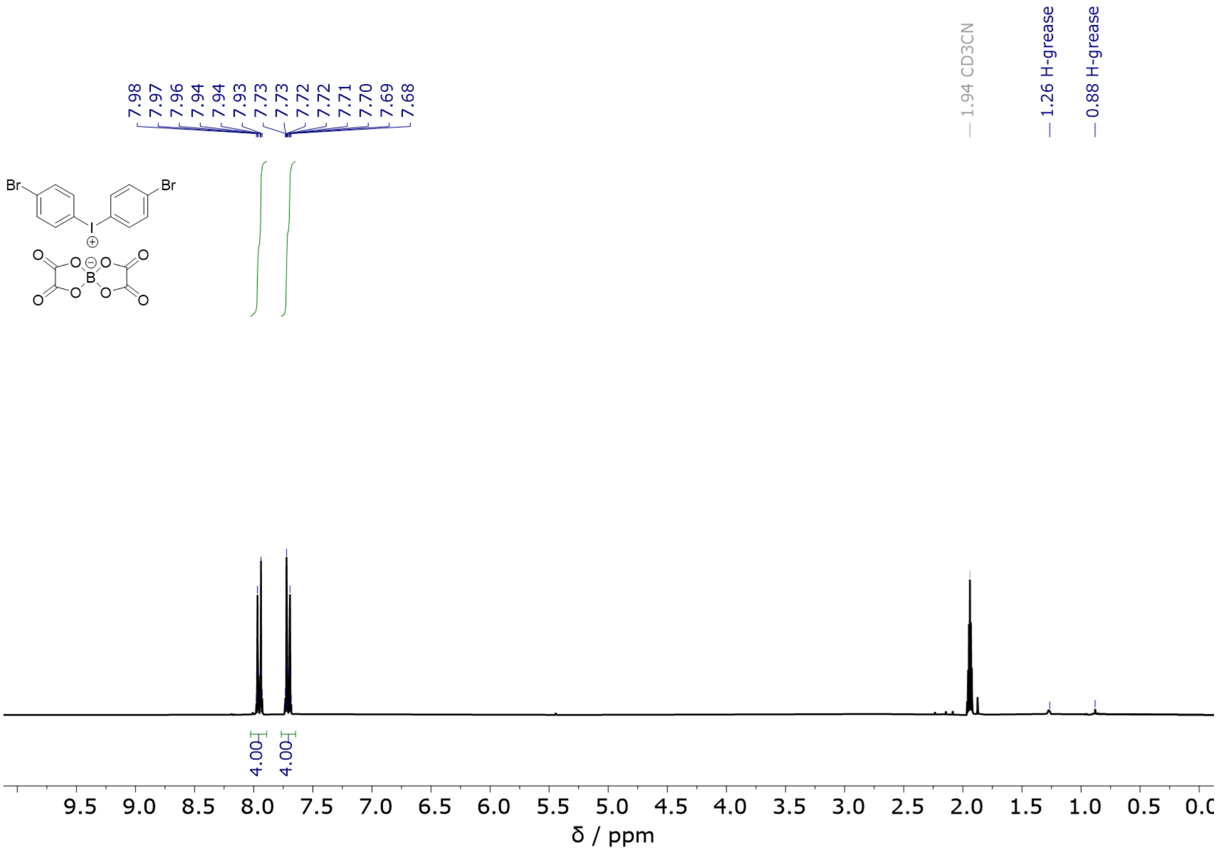

^{11}B NMR spectrum (96 MHz, CD_3CN) of bis(4-bromophenyl)iodonium bis(oxalato)borate (**7**).

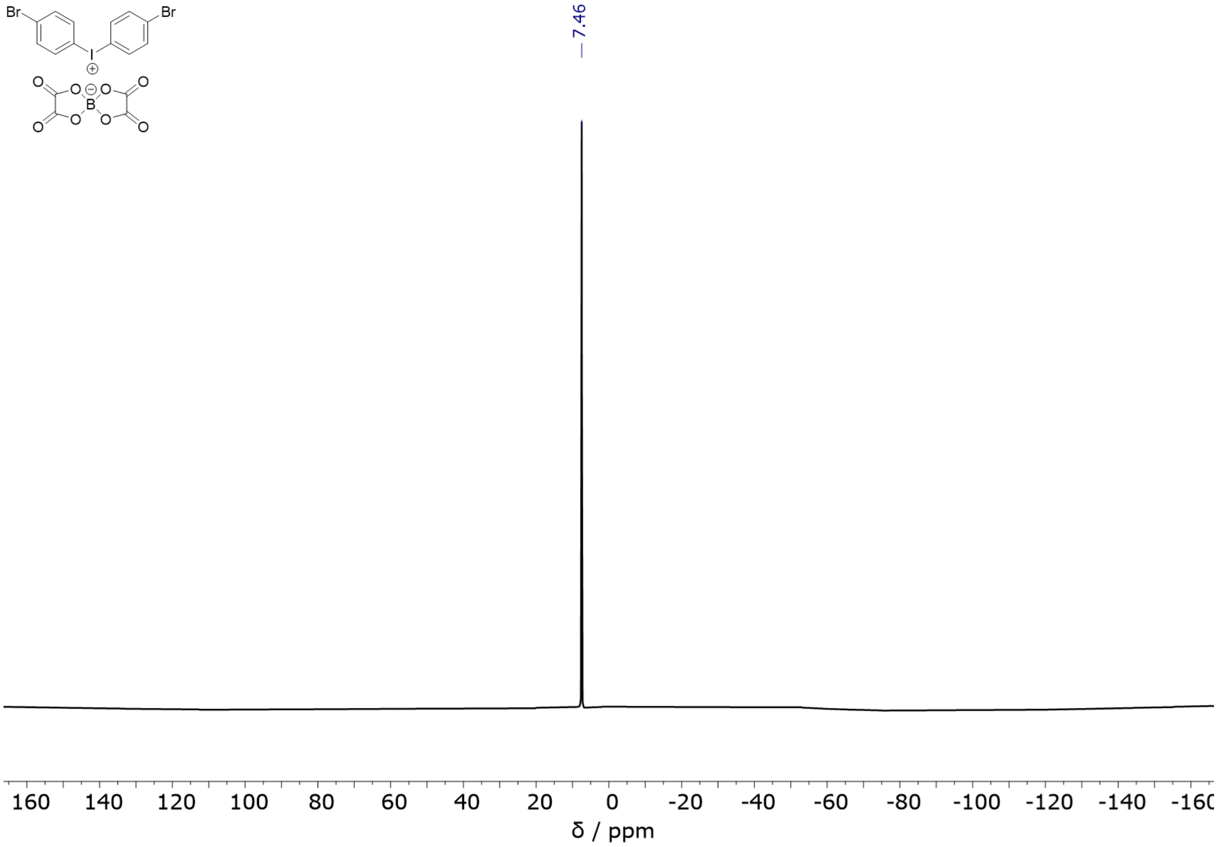

^{13}C{^{1}H} NMR spectrum (75 MHz, CD_3CN) of bis(4-bromophenyl)iodonium bis(oxalato)borate (**7**).

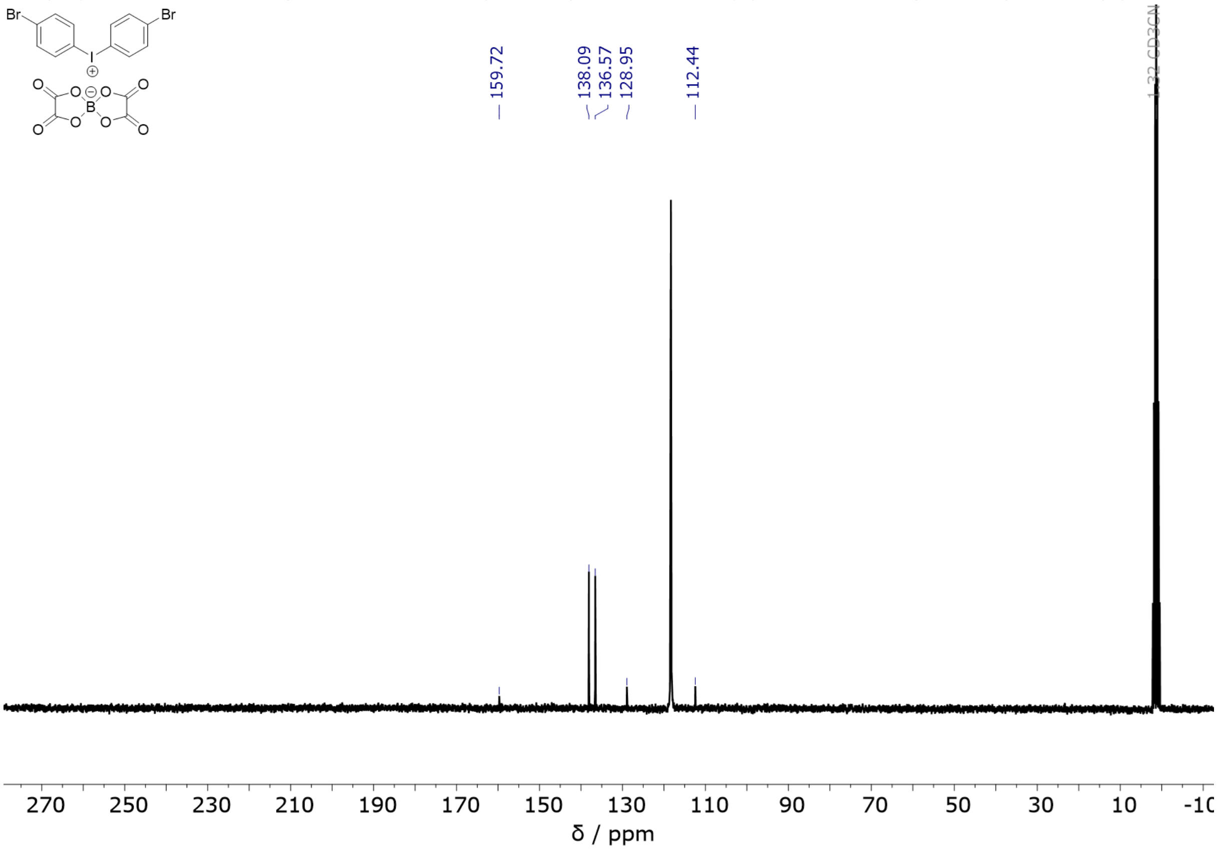

^{1}H NMR spectrum (300 MHz, CD_2Cl_2) of [1,1'-biphenyl]-4-carbaldehyde (**9a**).

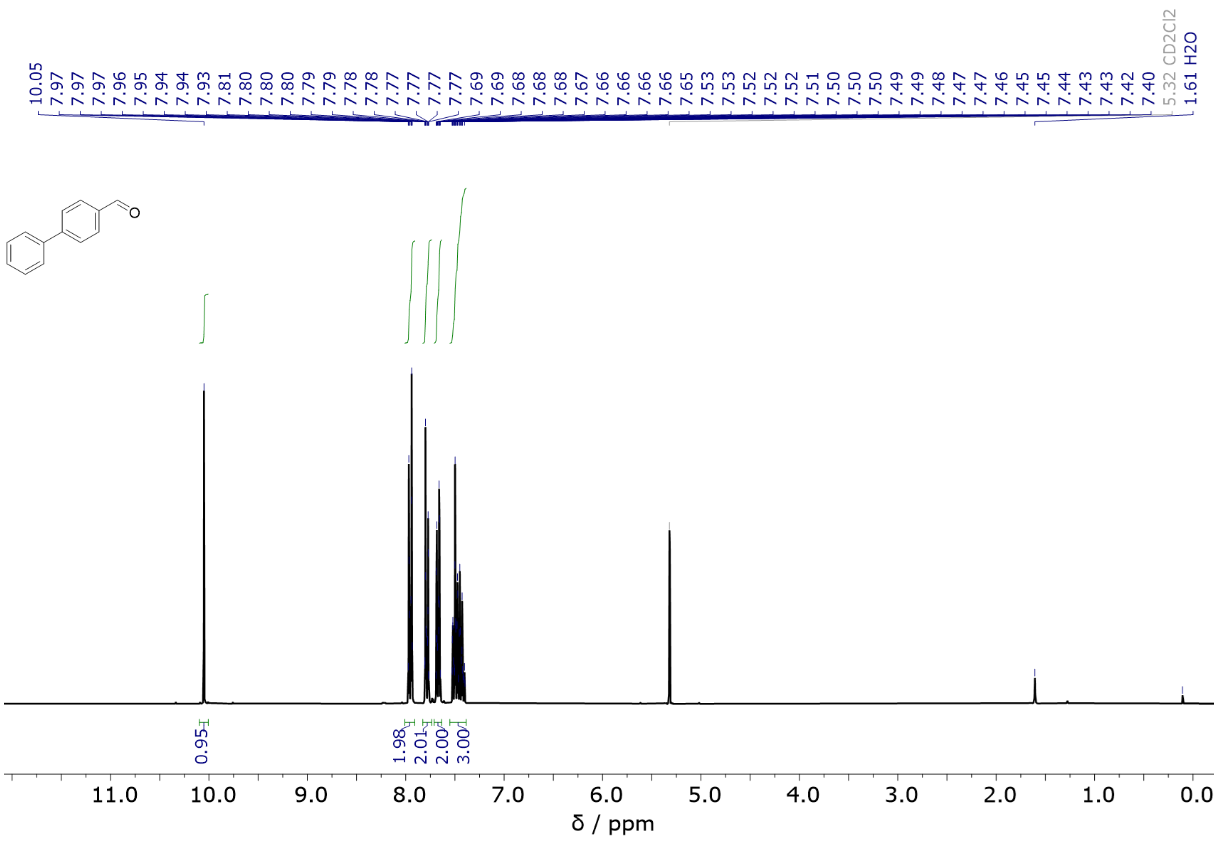

^{13}C{^{1}H} NMR spectrum (75 MHz, CD_2Cl_2) of [1,1'-biphenyl]-4-carbaldehyde (**9a**).

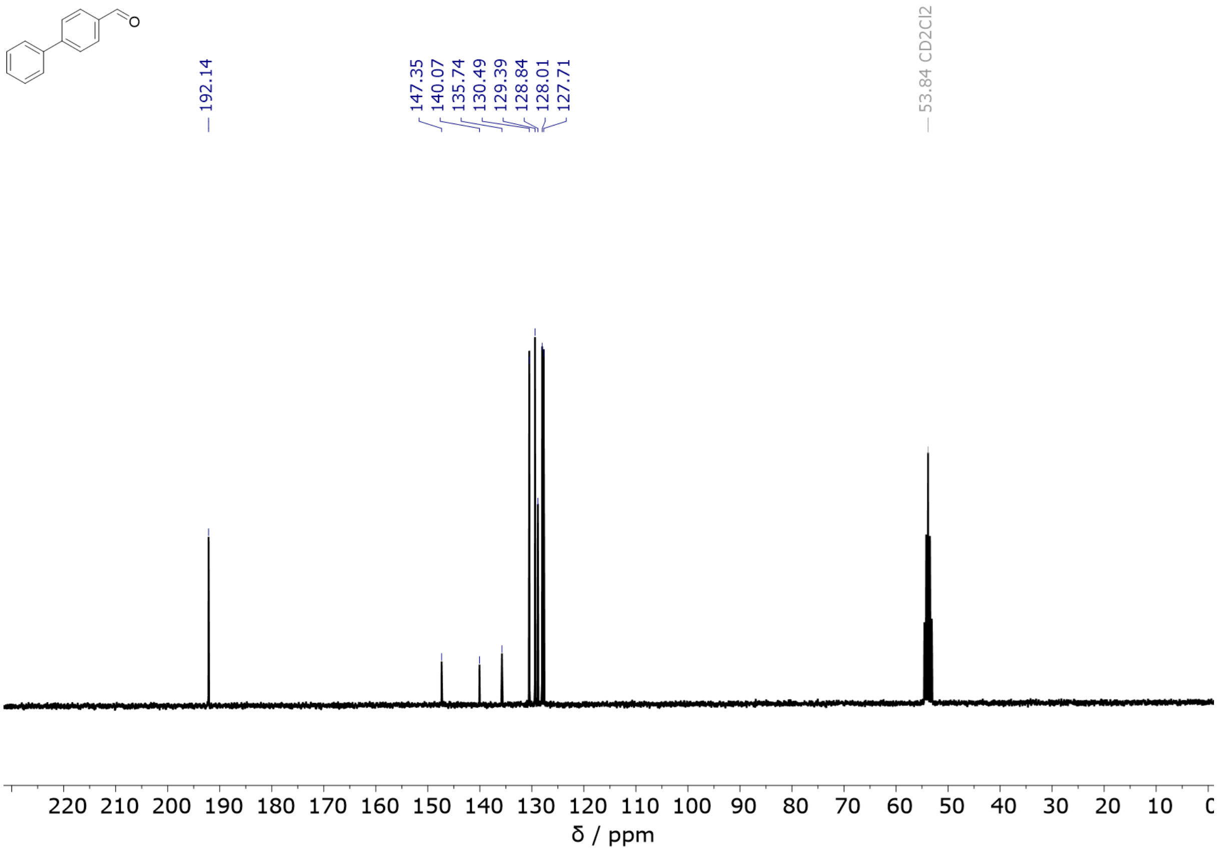

^{1}H NMR spectrum (300 MHz, CD_2Cl_2) of 2-naphthaldehyde (**9b**).

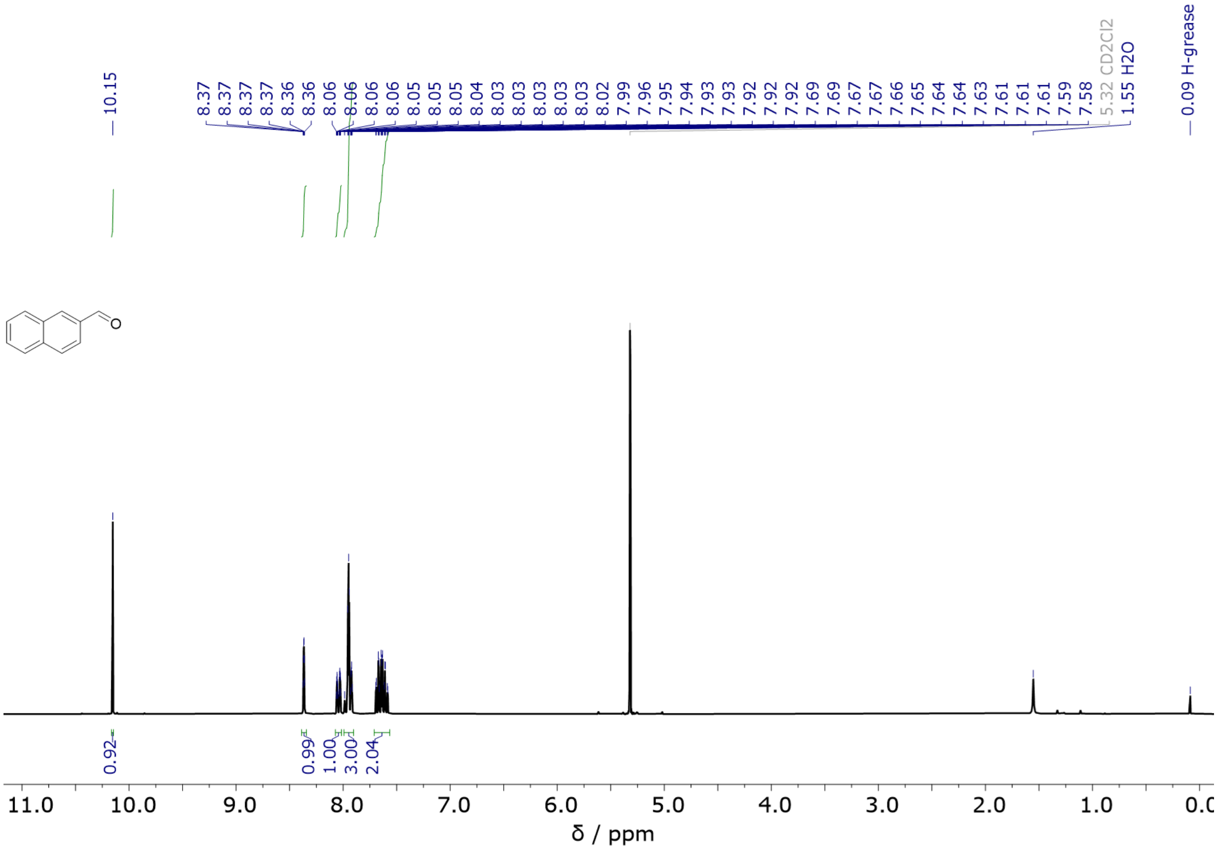

$^{13}C\{^{1}H\}$ NMR spectrum (75 MHz, CD_2Cl_2) of 2-naphthaldehyde (**9b**).

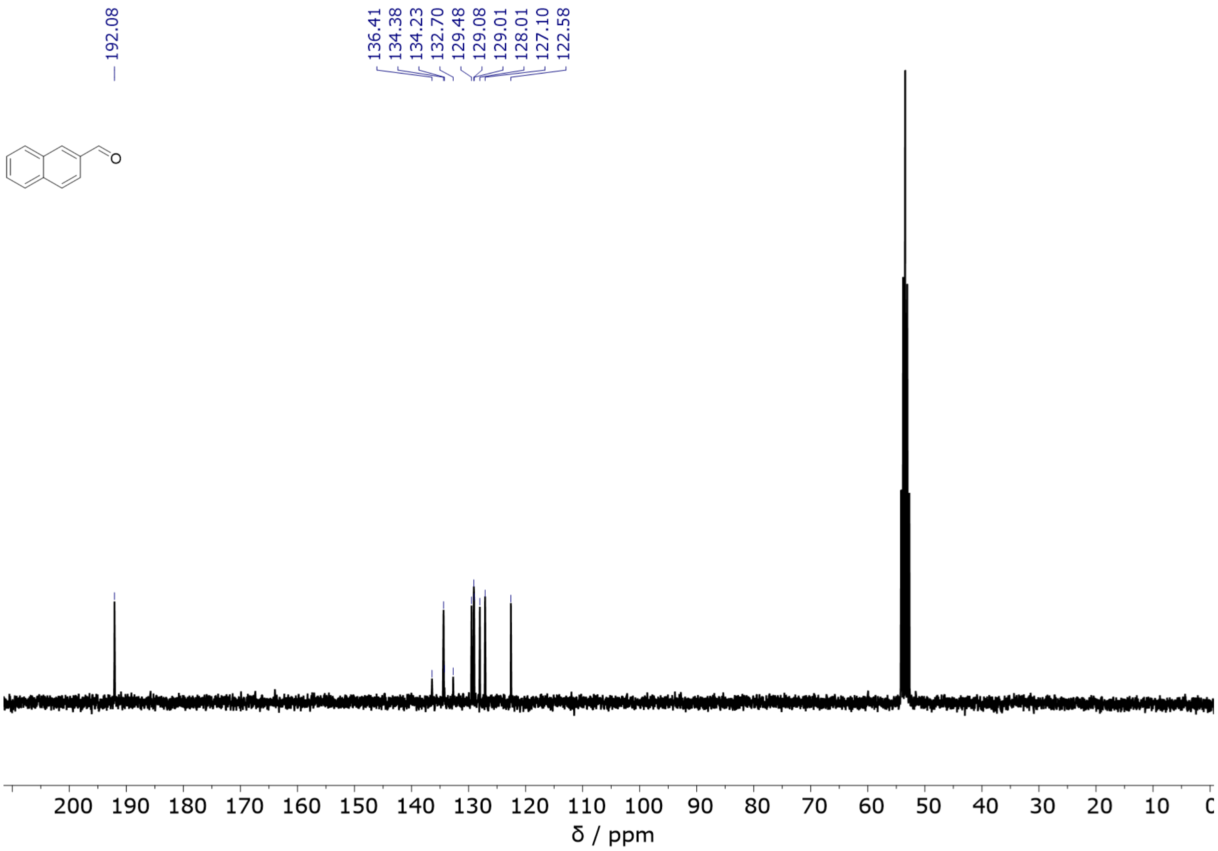

^{1}H NMR spectrum (400 MHz, CD_3CN) of tetraethylammonium bis(oxalato)borate (Et_4NBOB).

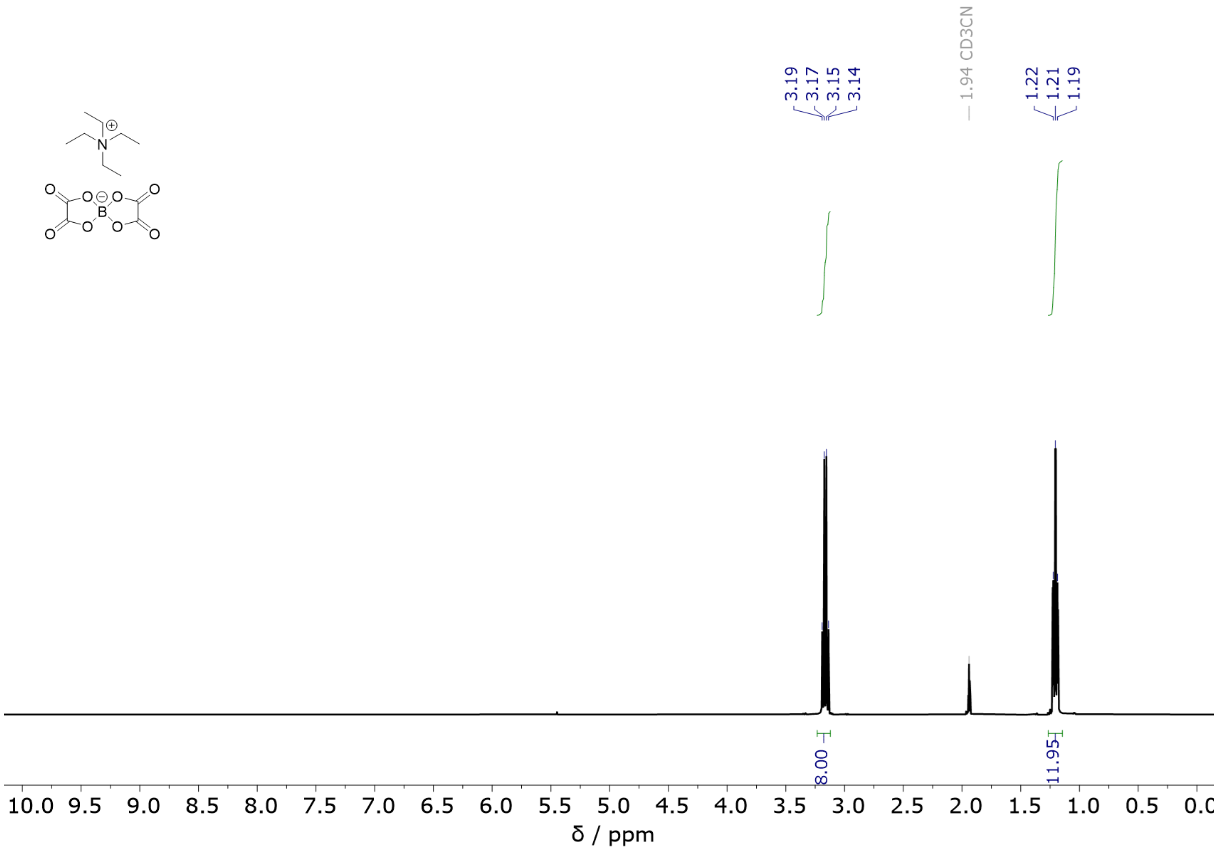

^{11}B NMR spectrum (128 MHz, CD_3CN) of tetraethylammonium bis(oxalato)borate (Et_4NBOB).

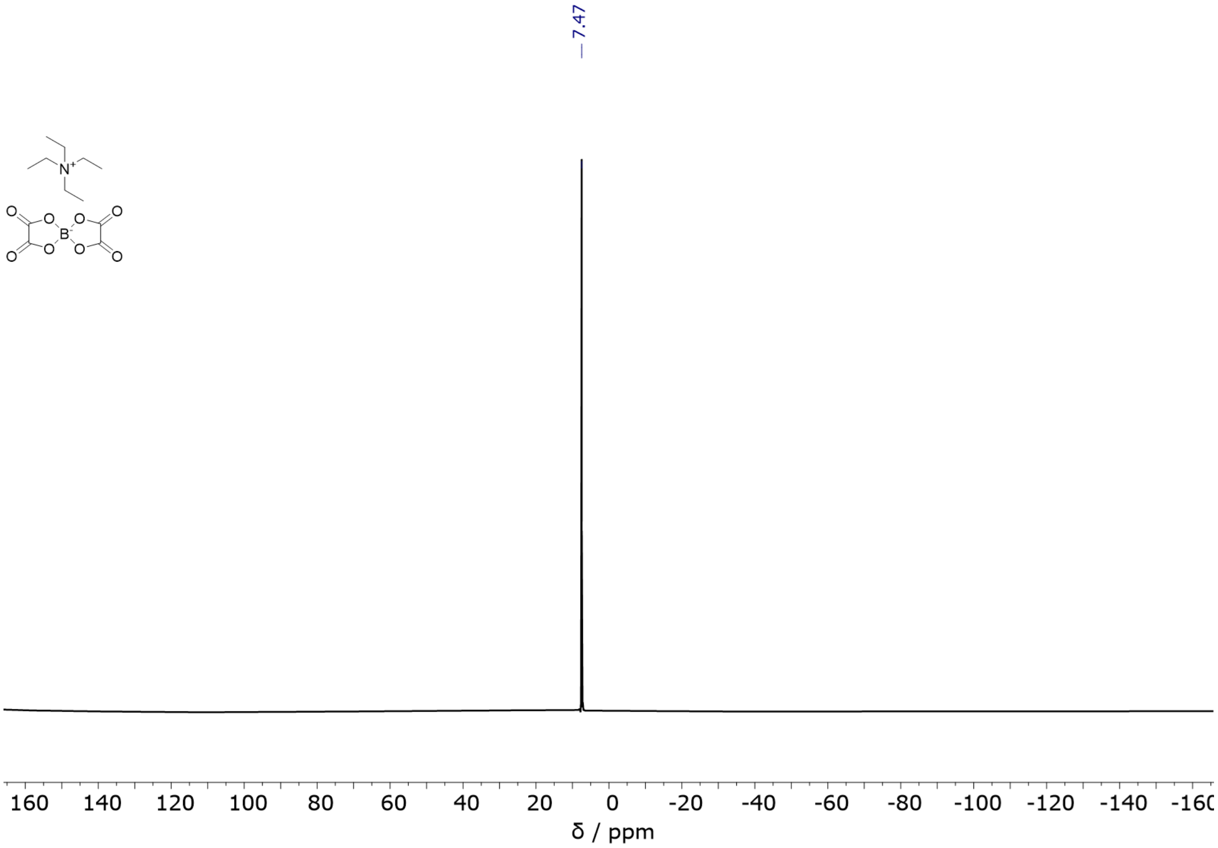

$^{13}C\{^{1}H\}$ NMR spectrum (101 MHz, CD_3CN) tetraethylammonium bis(oxalato)borate (Et_4NBOB).

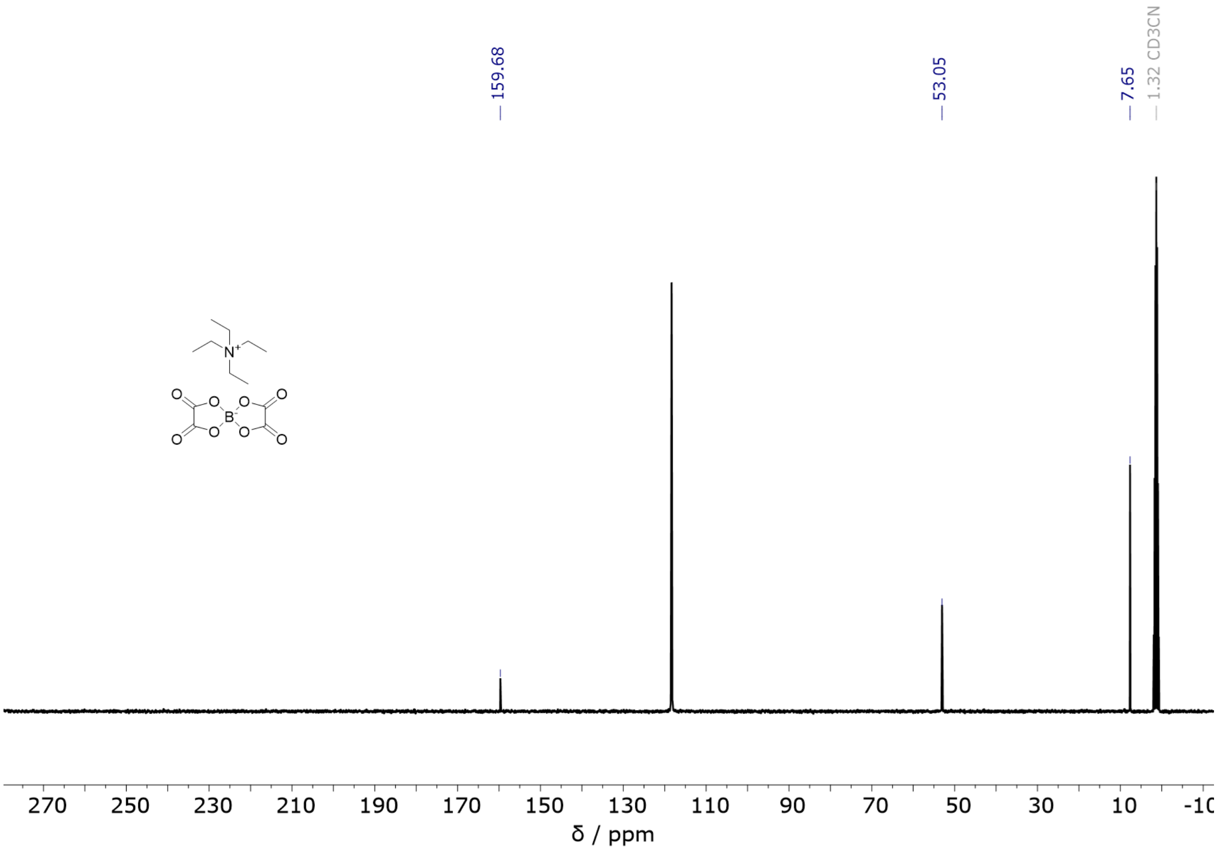

^{1}H NMR spectrum (300 MHz, CD_2Cl_2) of diphenylmethanol (**11**).

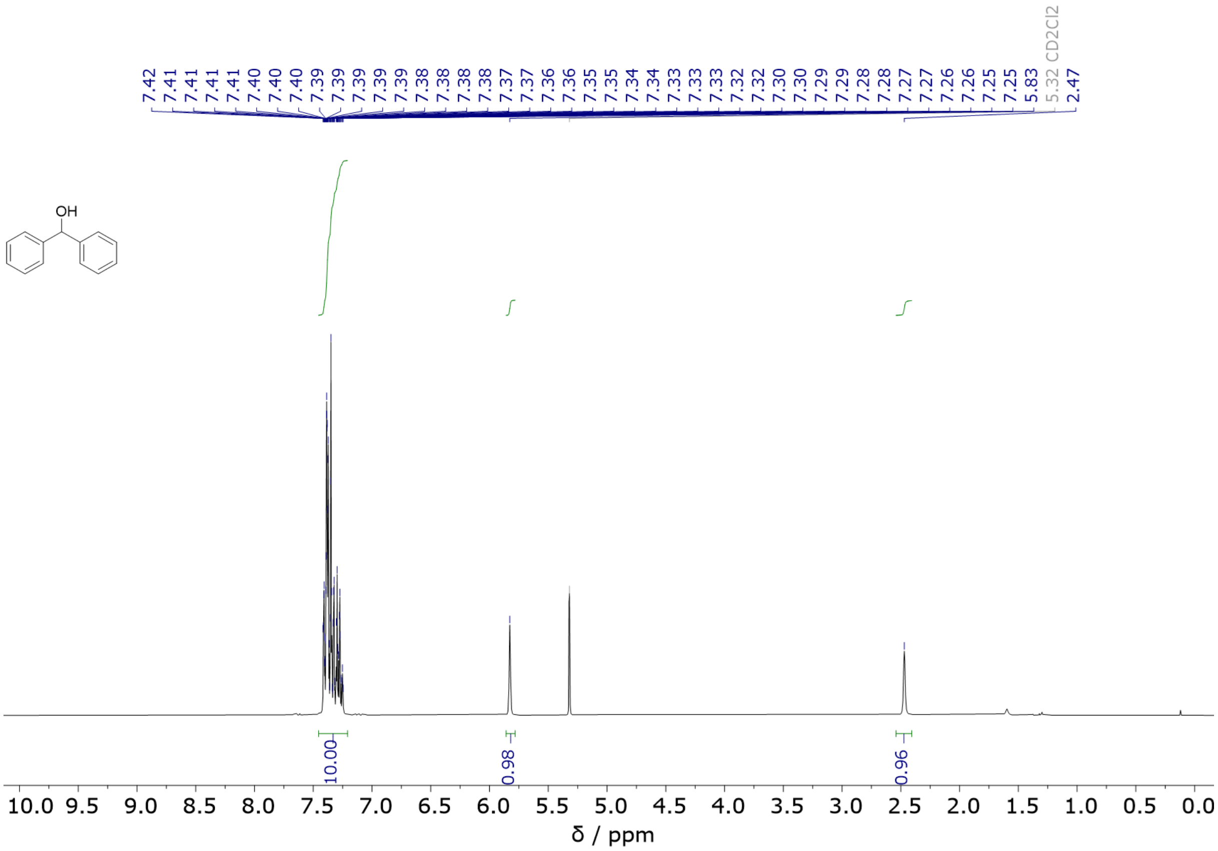

^{13}C{^{1}H} NMR spectrum (75 MHz, CD_2Cl_2) of diphenylmethanol (**11**).

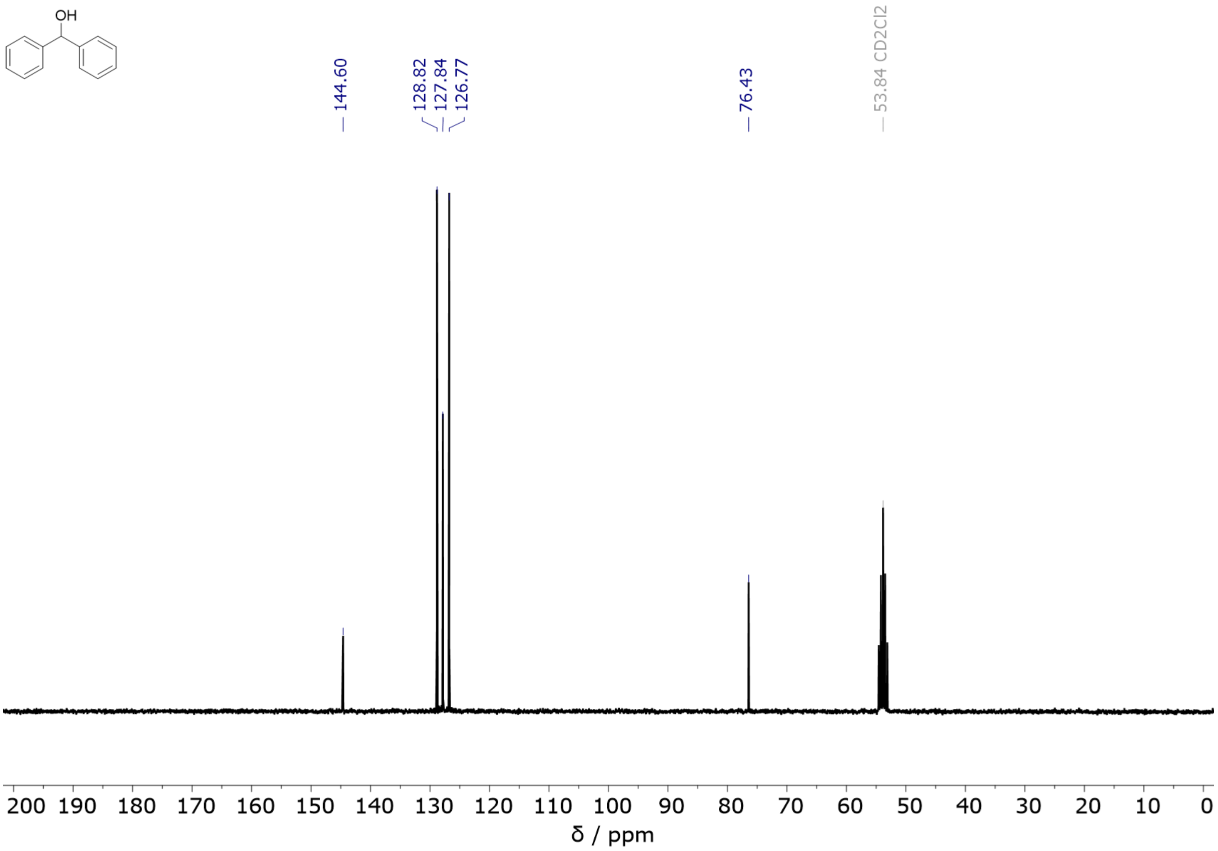

4.2 Quantification of product yields by ^{1}H NMR spectroscopy

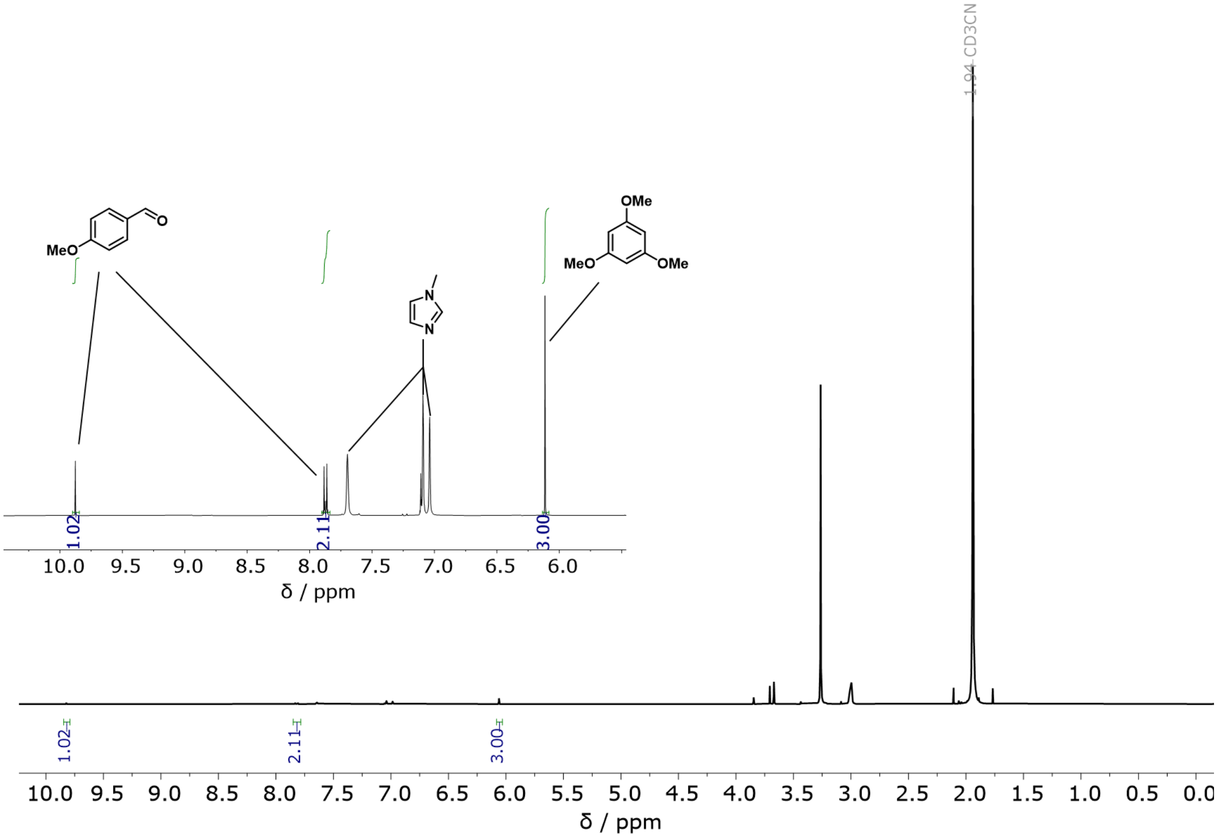

Figure S16. ^{1}H NMR spectrum of crude electrolysis mixture from TEMPO-mediated oxidation of *p*-methoxybenzyl alcohol (inset: characteristic region of aldehyde proton signals).

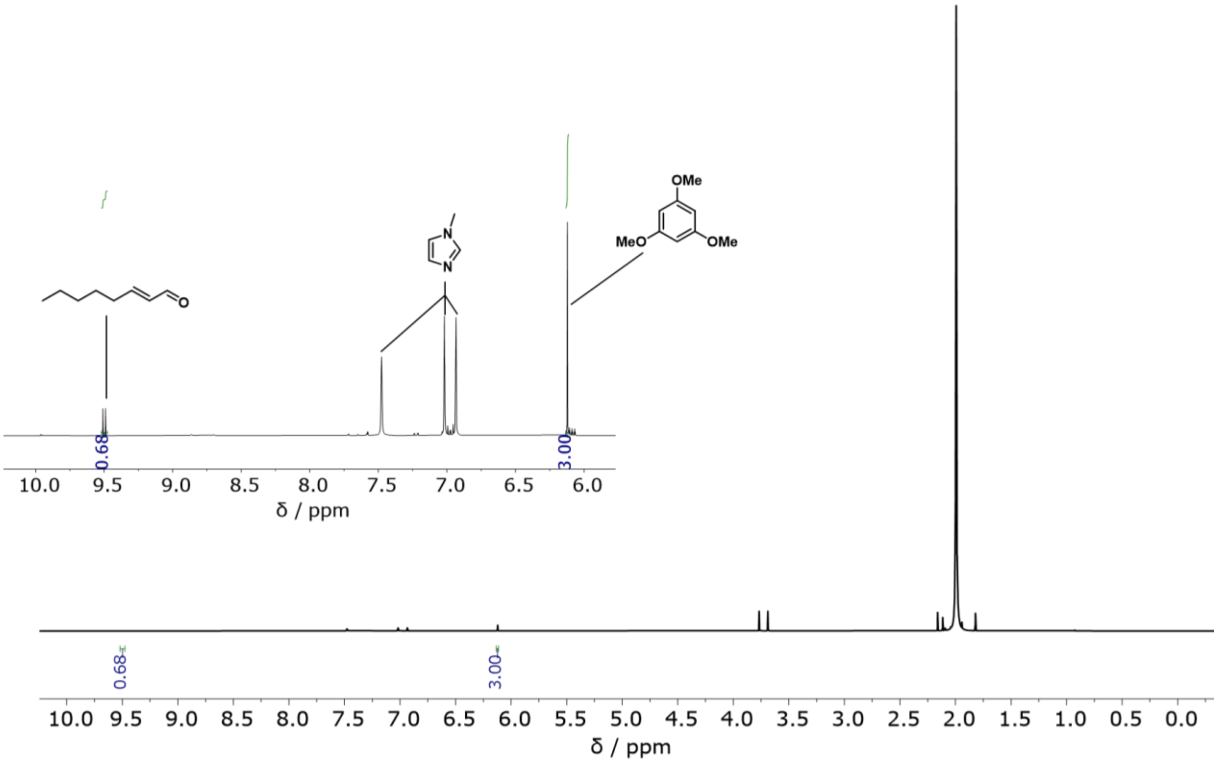

Figure S17. ^{1}H NMR spectrum of crude electrolysis mixture from TEMPO-mediated oxidation of (*E*)-2-octen-1-ol (inset: characteristic region of aldehyde proton signals).

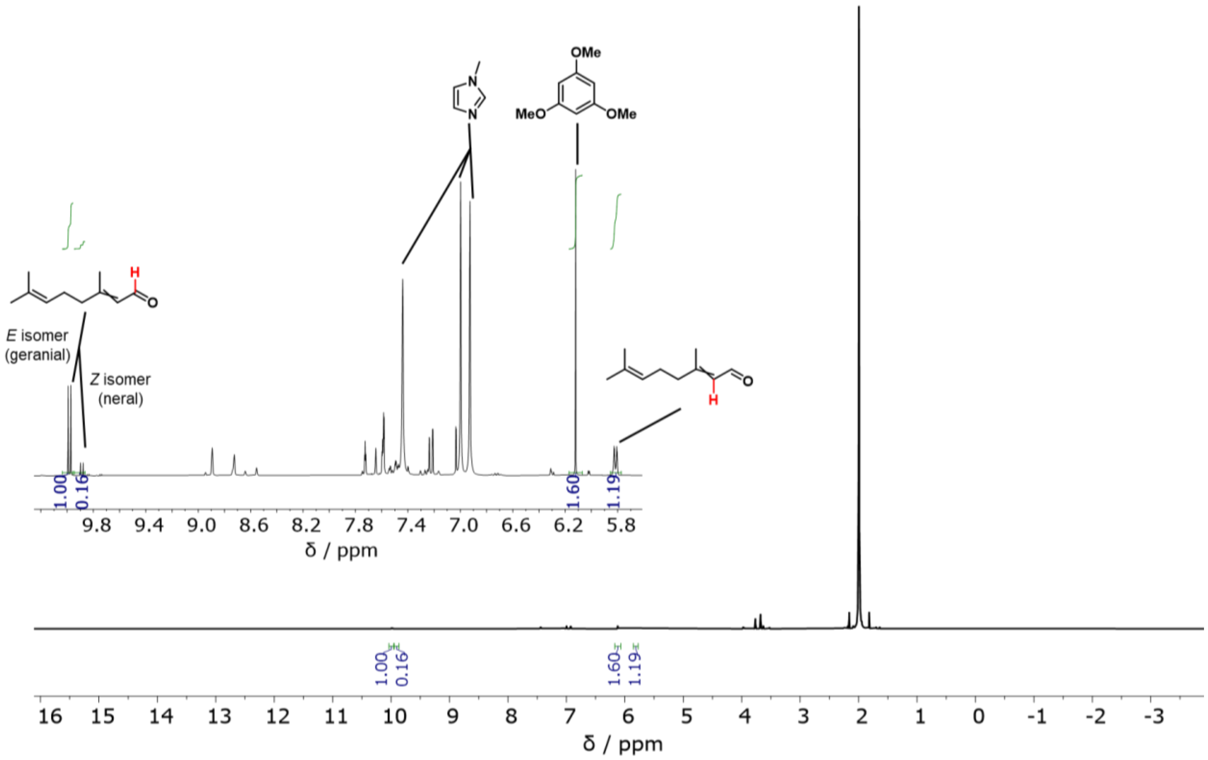

Figure S18. ^{1}H NMR spectrum of crude electrolysis mixture from TEMPO-mediated oxidation of geraniol (inset: characteristic region of aldehyde proton signals).

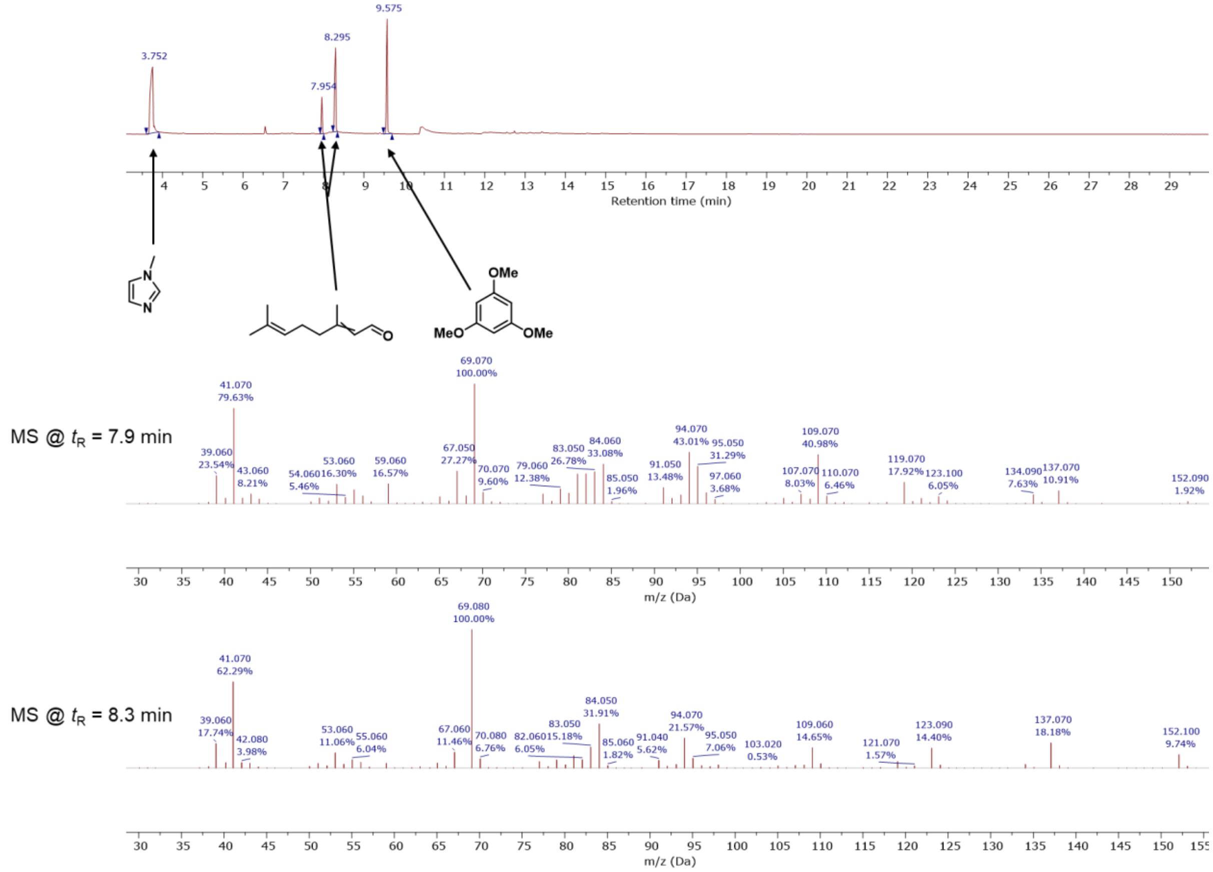

Figure S19. Gas chromatogram (top) and corresponding mass spectra (bottom) obtained from analysis of the reaction mixture after completed TEMPO-catalyzed electro-conversion of geraniol.

Starting from commercially available geraniol with a geranial content of approx. 1.1% and a neral content of approx. 0.6% (according to ^{1}H NMR analysis), the two isomers (*E*)-3,7-dimethylocta-2,6-dienal (geranial) and (*Z*)-3,7-dimethylocta-2,6-dienal (neral) were detected in a 6.2 : 1 ratio in the crude electrolysis mixture (see Figure S18). Assignment of the ^{1}H NMR signals was achieved based on the literature.[8] *E*-*Z* Isomerization was confirmed qualitatively by GC-MS analysis (see Figure S19). The 81% ^{1}H NMR yield (Scheme 2) is an average value calculated based on two trials.

8 Chemical shifts of aldehyde protons of *E*/*Z* isomers were assigned according to the data reported the following ref.: M. Schmidt, V. Huber, D. Tourand, W. Kunz, *Molecules*, **2024**, *29*, 294.

5. Green metrics estimated for the synthesis of 11

The process mass intensity (PMI), the solvent intensity (PMI_{solv}), and PMI_{RRC} were calculated according to the following equation[9] and are summarized in Table 1 (see manuscript).

$$PMI = \frac{m_{reactants} + m_{reagents} + m_{catalyst}}{m_{product}} + \frac{m_{solvent}}{m_{product}} = PMI_{RRC} + PMI_{solv}$$

Table S6. Summary of the parameters used for calculation of PMI, PMI_{RRC}, and PMI_{solv} values for the conversion of **10** to **11** on a 5.0 mmol scale in acetonitrile (see Table 1 in manuscript).[10]

Parameter	Benzophenone (**10**)	DABCO	Et_4NBOB	$(Ph)_2CHOH$ (**11**)	CH_3CN
MW [g mol^{-1}]	182.22	112.18	317.10	184.24	41.05
V [mL]	-	-	-	-	15
ρ [g mL^{-1}]	-	-	-	-	0.78
n [mmol]	5.0	15.0	1.125	4.5	-
c [mmol L^{-1}]	333	1000	75		
m [g]	0.911	1.68	0.357	0.829	11.70
Isolated yield [%]				90	

Table S7. Summary of the parameters used for calculation of PMI, PMI_{RRC}, and PMI_{solv} values for the conversion of **10** to **11** on a 5.0 mmol scale in PC/DMC (4:1) (see Table 1 in manuscript).[10]

Parameter	Benzophenone (**10**)	DABCO	Et_4NBOB	$(Ph)_2CHOH$ (**11**)	PC	DMC
MW [g mol^{-1}]	182.22	112.18	317.10	184.24	102.09	90.08
V [mL]	-	-	-	-	-	-
ρ [g mL^{-1}]	-	-	-	-	1.21	1.07
n [mmol]	5.0	15.0	1.125	3.45		
c [mmol L^{-1}]	333	1000	75			
m [g]	0.911	1.683	0.357	0.636	18.05	
Isolated yield [%]				69		

[9] E. R. Monteith, P. Mampuys, L. Summerton, J. H. Clark, B. U. W. Maes, C. R. McElroy, *Green Chem.*, **2020**, 22, 123-135.

[10] The mass of the product was extrapolated taking into account the aliquot removed from the electrolyte solution for product quantification *via* ^{1}H NMR analysis.

Table S8. Summary of the parameters used for calculation of PMI, PMI_{RRC}, and PMI_{solv} values for electrochemical conversion of **10** to **11** in DMF.[11]

Parameter	Benzophenone (**10**)	DABCO	*n*Bu_4NBF_4	$(Ph)_2CHOH$ (**11**)	DMF
MW [g mol^{-1}]	182.22	112.18	329.27	184.24	73.10
V [mL]	-	-	-	-	5
ρ [g mL^{-1}]	-	-	-	-	0.95
n [mmol]	0.3	0.9	0.25	-	-
c [mmol L^{-1}]	60	180	50		
m [g]	0.055	0.101	0.082	0.044	4.75
Isolated yield [%]				79	

Table S9. Summary of the parameters used for calculation of PMI, PMI_{RRC}, and PMI_{solv} values for non-electrochemical ketone reduction in ethanol using $NaBH_4$ as reducing reagent.[12]

Parameter	Benzophenone (**10**)	$NaBH_4$	$(Ph)_2CHOH$ (**11**)	EtOH
MW [g mol^{-1}]	182.22	37.83	184.24	46.07
V [mL]	-	-	-	50
ρ [g mL^{-1}]	-	-	-	0.79
n [mmol]	5.5	3.6	-	-
c [mmol L^{-1}]	110	72		
m [g]	1.000	0.137	1.010	39.50
Isolated yield [%]			>99	

[11] L. Wang, X. Zhang, R. Y. Xia, C. Yang, L. Guo, W. J. Xia, *Synlett* **2022**, *33*, 1302-1308.
[12] J. Desroches, P. A. Champagne, Y. Benhassine, J.-F. Paquin, *Org. Biomol. Chem.* **2015**, *13*, 2243-2246.
